# Catalytic transfer hydrogenation of N_2_ to NH_3_ via a photoredox catalysis strategy

**DOI:** 10.1126/sciadv.ade3510

**Published:** 2022-10-26

**Authors:** Christian M. Johansen, Emily A. Boyd, Jonas C. Peters

**Affiliations:** Division of Chemistry and Chemical Engineering, California Institute of Technology, Pasadena, CA 91125, USA.

## Abstract

Inspired by momentum in applications of reductive photoredox catalysis to organic synthesis, photodriven transfer hydrogenations toward deep (>2 e^−^) reductions of small molecules are attractive compared to using harsh chemical reagents. Noteworthy in this context is the nitrogen reduction reaction (N_2_RR), where a synthetic photocatalyst system had yet to be developed. Noting that a reduced Hantzsch ester (HEH_2_) and related organic structures can behave as 2 e^−^/2 H^+^ photoreductants, we show here that, when partnered with a suitable catalyst (Mo) under blue light irradiation, HEH_2_ facilitates delivery of successive H_2_ equivalents for the 6 e^−^/6 H^+^ catalytic reduction of N_2_ to NH_3_; this catalysis is enhanced by addition of a photoredox catalyst (Ir). Reductions of additional substrates (nitrate and acetylene) are also described.

## Teaser

Photoinduced Mo-catalyzed transfer hydrogenation of N_2_ to NH_3_ from a Hantzsch ester (HEH_2_) with and without a photoredox catalyst.

## INTRODUCTION

**Fig. 1.**
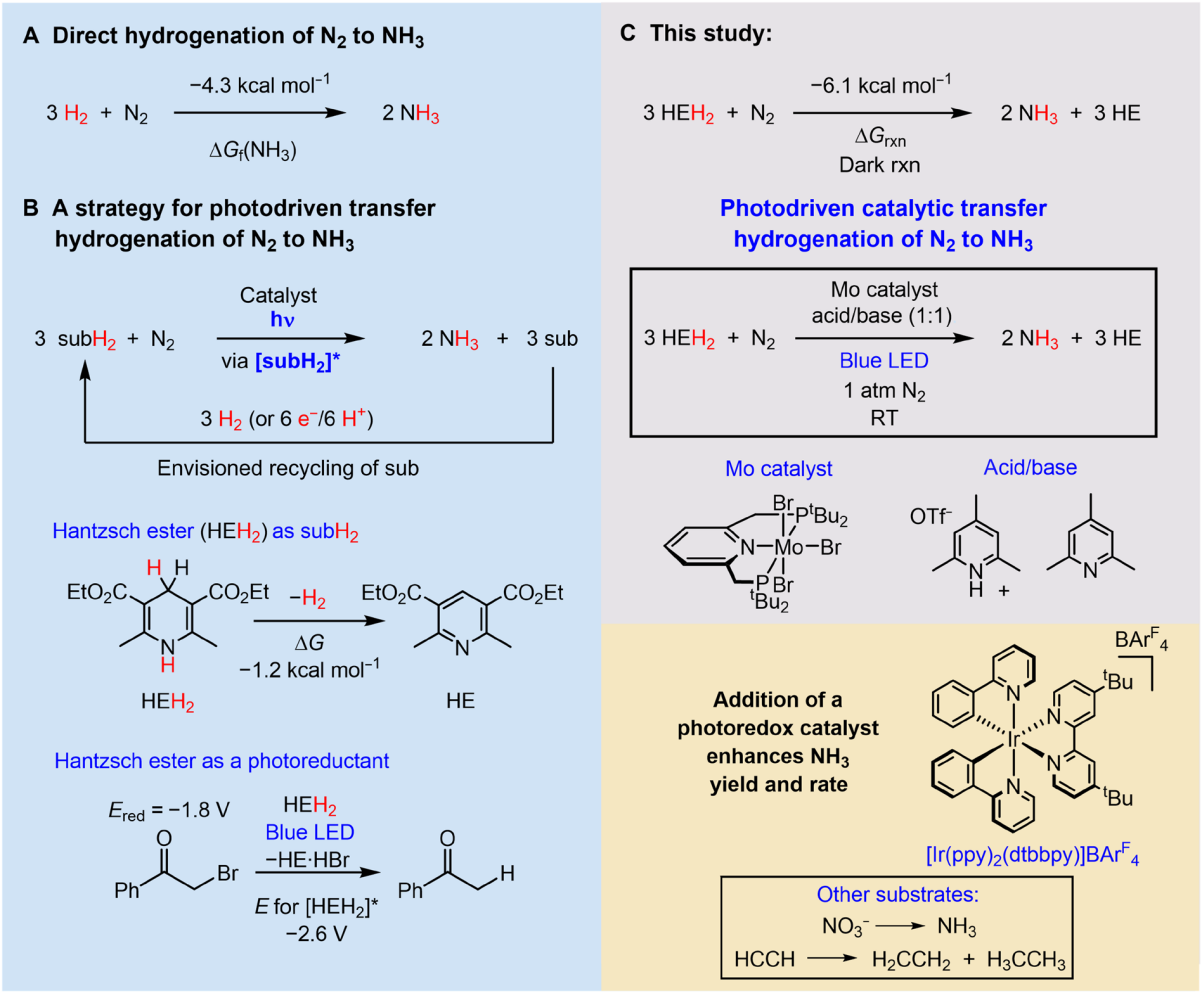
Thermodynamics and strategies for hydrogenation of N_2_. (**A**) Thermodynamics of hydrogenation of N_2_ to NH_3_. (**B**) Schematic of an overall design for light-driven transfer hydrogenation of N_2_, chemical structure of the Hantzsch ester used in this study (HEH_2_), and representative reduction of α-bromoacetophenone. (**C**) Net stoichiometry and estimated driving force of transfer hydrogenation from HEH_2_ to N_2_, forming NH_3_; photodriven (blue LED) process described in this study, in the absence and presence of a photoredox catalyst. All thermochemical values are given in MeCN at 25°C with ferrocenium/ferrocene (Fc^+/0^) as the reference potential. RT, room temperature.

Multielectron reductive transformations of small-molecule substrates (e.g., N_2_, CO_2_, and NO_3_^−^) are challenging to mediate in homogeneous catalysis and most typically require considerable energy input via harsh chemical reagents and/or conditions to be driven forward. The nitrogen reduction reaction (N_2_RR) offers a case in point; substantial progress has now been made in molecular catalyst design, but substantial overpotentials are generally needed to observe the NH_3_ product (*1*–*3*). For nitrogen reduction (N_2_R), kinetic challenges also prevail for enzymatic and heterogeneous catalysis that require substantial energy inputs, via adenosine triphosphate hydrolysis for the former and high temperature and pressure or electrochemical overpotential for the latter (*4*–*6*), despite a thermally favorable Gibbs free energy of formation, Δ*G*_f_(NH_3_) (Fig. 1A).

The organometallic catalysis field has pursued photochemical strategies as a means of driving small-molecule reductions, with considerable success being achieved for CO_2_ reduction (CO_2_R; typically by 2 e^−^/2 H^+^) as the target transformation (*7*, *8*). These strategies are still challenged by the widespread use of sacrificial donors whose oxidation products are not readily recycled. While design schemes are envisaged to someday couple photodriven CO_2_R catalysis with water oxidation, photodriven transfer hydrogenation using a suitable precatalyst offers an approach to reductive small-molecule catalysis, especially if the net H_2_ donor (subH_2_; Fig. 1B) derives from a structure that can be efficiently recycled, for example, via hydrogenation or electrochemically.

Reduced Hantzsch esters (HEH_2_; Fig. 1B) and chemically related structures (e.g., reduced acridine and phenanthridine) have been explored for thermally and photochemically driven reductive hydride (H^−^; NADH-like) and H atom transfers in organic synthesis (*9*). Moreover, they are highlighted for their chemical (and electrochemical) recyclability via net hydrogenation of the spent pyridine-type oxidation product (*10*, *11*). Whereas the types of transformations they participate in are most typically two-electron processes, they are also tempting to explore for deeper multielectron reductions of the type pursued in small-molecule reductive catalysis. Focusing on N_2_R (*12*), we noted that despite long known and still debated studies of photocatalytic nitrogen fixation using semiconductors (*13*–*15*), and photodriven N_2_R mediated by nitrogenase coupled with CdS (*16*, *17*), as yet, there were no examples of photochemically driven catalytic N_2_R using well-defined molecular systems. Hence, photoinduced N_2_R via transfer hydrogenation from a Hantzsch ester or related donor, which requires the donors to engage in successive transfers to mediate a deep 6 e^−^/6 H^+^ reduction process, provides an excellent test case of this strategy for small-molecule substrates.

Considering thermodynamic parameters relevant to the aforementioned goals, in its ground state, the first C─H bond dissociation free energy (BDFE_C─H_) of HEH_2_ is 62.3 kcal mol^−1^ in MeCN at 25°C (all following thermochemical values are defined at these conditions), which is not weak enough to bimolecularly liberate H_2_ (*18*). Photoexcitation of HEH_2_, however, renders an excited state that is highly reducing [*E*_ox_ for [HEH_2_]* is ~ −2.6 V versus ferrocenium/ferrocene (Fc^+/0^)] (*19*, *20*). Photodriven [blue light-emitting diode (LED)] reduction of α-bromoacetophenone to acetophenone by HEH_2_ illustrates its capacity to deliver an H_2_ equivalent (Fig. 1B) (*19*). For a dark N_2_R reaction, we estimate the overpotential for reduction of N_2_ by HEH_2_ to generate NH_3_ as 1.8 kcal mol^−1^ [ΔΔ*G*_f_(NH_3_); Fig. 1C]. Using light (blue LED), we show here that it is indeed possible to catalyze photoinduced transfer hydrogenation from HEH_2_ to N_2_ using Nishibayashi’s molybdenum precatalyst (Fig. 1C) (*21*) at atmospheric pressure and 23°C. The inclusion of an Ir photoredox catalyst (Fig. 1C) within this system, while not necessary for turnover, enhances the yields and rates of NH_3_ generation.

For our present catalysis system, we noted that a photoreduction step from the excited state of HEH_2_, [HEH_2_]*, liberates the ground-state radical cation HEH_2_^•+^, which is a sufficiently strong oxidant (*E*_red_ = 0.48 V versus Fc^+/0^) to be deleterious to N_2_R (*18*). We therefore reasoned that inclusion of a base to deprotonate HEH_2_^•+^ (p*K*_a_ ~ −1) would be prudent (*18*). However, the presence of a moderate Brønsted acid is typically required for chemically driven N_2_R, suggesting that a buffered system might be needed. A collidine/collidinium [abbreviated as Col/[ColH]^+^; Col (2,4,6-trimethylpyridine)] mixture was chosen as Col will readily deprotonate HEH_2_^•+^, while [ColH]^+^, with a p*K*_a_ of 15 in MeCN (*22*), has been previously shown to be compatible with chemically driven N_2_R using (PNP)MoBr_3_ as a precatalyst {PNP [2,6-bis(di-*tert*-butylphosphinomethyl)pyridine]} with (Cp*)_2_Co [*E*_1/2_(Co^III/II^) = −1.91 V; Cp* (pentamethylcyclopentadienyl)] as the reductant (*21*, *23*).

## RESULTS AND DISCUSSION

**Fig. 2.**
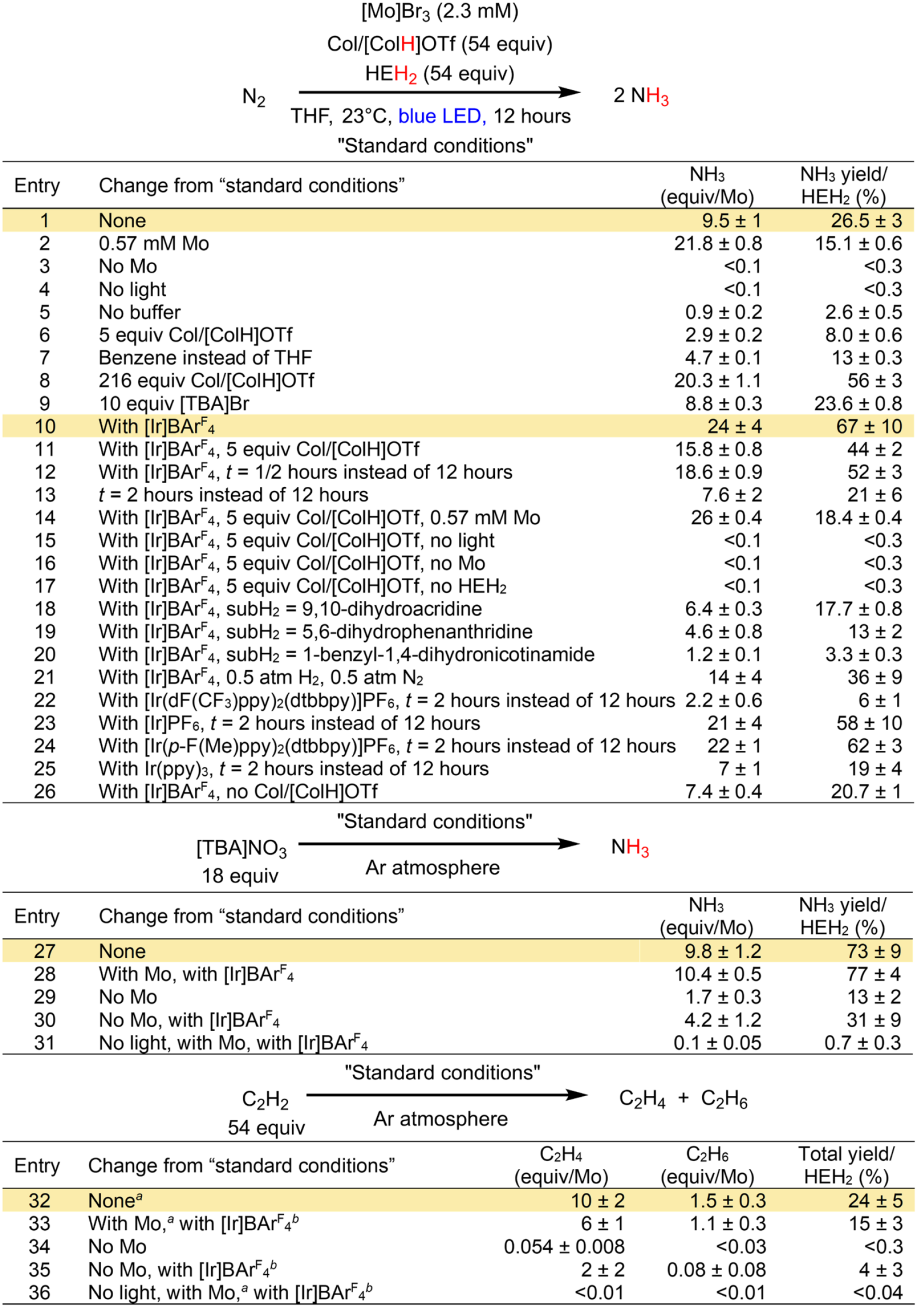
Catalytic yields for photodriven transfer hydrogenation of N_2_ to NH_3_, NO_3_^−^ to NH_3_, and acetylene to ethylene and ethane. Reactions performed with 2.3 mM [Mo]Br_3_ concentration, using a single 34-W Kessel H150 blue lamp unless otherwise noted. All yields reported are an average of at least two runs. All runs with Ir used 2.3 mM photosensitizer loading unless otherwise noted. *^a^*3.6 mM [Mo]Br_3_. *^b^*3.6 mM [Ir]BAr^F^_4_. [Ir], [Ir(ppy)_2_(dtbbpy)]^+^; ppy, 2-phenylpyridinyl; dtbbpy, 4,4′-di-*tert*-butyl-2,2′-bipyridine; BAr^F^_4_, tetrakis(3,5-bis(trifluoromethyl)phenyl)borate; dF(CF_3_)ppy, 5-trifluoromethyl-2-(3,5-difluoro-phenyl)-pyridine; *p*-F(Me)ppy, 5-methyl-2-(5-fluoro-phenyl)-pyridine; PF_6_^−^, hexafluorophosphate.

We find that [Mo]Br_3_ (1 equiv at 2.3 mM) in the presence of 54 equiv each of HEH_2_, [ColH]OTf [OTf (triflate)], and Col in tetrahydrofuran (THF), under an N_2_ atmosphere and blue LED irradiation at 23°C for 12 hours, yields 9.5 ± 1 equiv of NH_3_/Mo (Fig. 2, entry 1). Assuming that HEH_2_ is a 2 e^−^ donor in this process provides an NH_3_ yield with respect to HEH_2_ of ~25%. Use of ^15^N_2_ confirmed N_2_ as the source of the NH_3_ produced (fig. S2). To cement this interpretation, using either ^15^N-labeled HEH_2_ or ^15^N-labeled Col/[ColH]OTf produced only ^14^NH_3_. Analysis of the organic products following catalysis revealed complete consumption of HEH_2_, with the fully oxidized Hantzsch ester pyridine (HE) as the major organic by-product, consistent with HEH_2_ acting as a 2 e^−^/2 H^+^ donor. We note that the yield of HE is ~90%; similarly, ~10% of the initial buffer loading is not recovered (fig. S7). In addition to HE and recovered buffer, a complex mixture of organic species is observed following catalysis. A major component of this mixture is generated independently via irradiation of HEH_2_ and buffer in the absence of metal catalysts (fig. S8), possibly as a result of light-induced reductive coupling as has been previously observed upon irradiation of HE in the presence of amine reductants (*24*). Another factor limiting NH_3_ selectivity per HEH_2_ concerns background hydrogen evolution under blue light irradiation (see fig. S10).

Higher yields of NH_3_ per Mo center could be obtained by decreasing the [Mo]Br_3_ loading (21.8 ± 0.8 equiv per Mo; entry 2), but with a loss in the yield of NH_3_ with respect to HEH_2_. The Mo catalyst and irradiation were required to generate NH_3_, and yields were substantially lower without the added buffer (entries 3 to 5). Attempts to use catalytic amounts of Col/[ColH]OTf (5 equiv per [Mo]Br_3_) substantially lowered the NH_3_ yields (entry 6). The reaction run in benzene instead of THF solvent remained catalytic but gave attenuated yields (4.7 ± 0.1 equiv of NH_3_/Mo; entry 7), likely because of the lower solubility of [ColH]OTf in benzene.

**Fig. 3.**
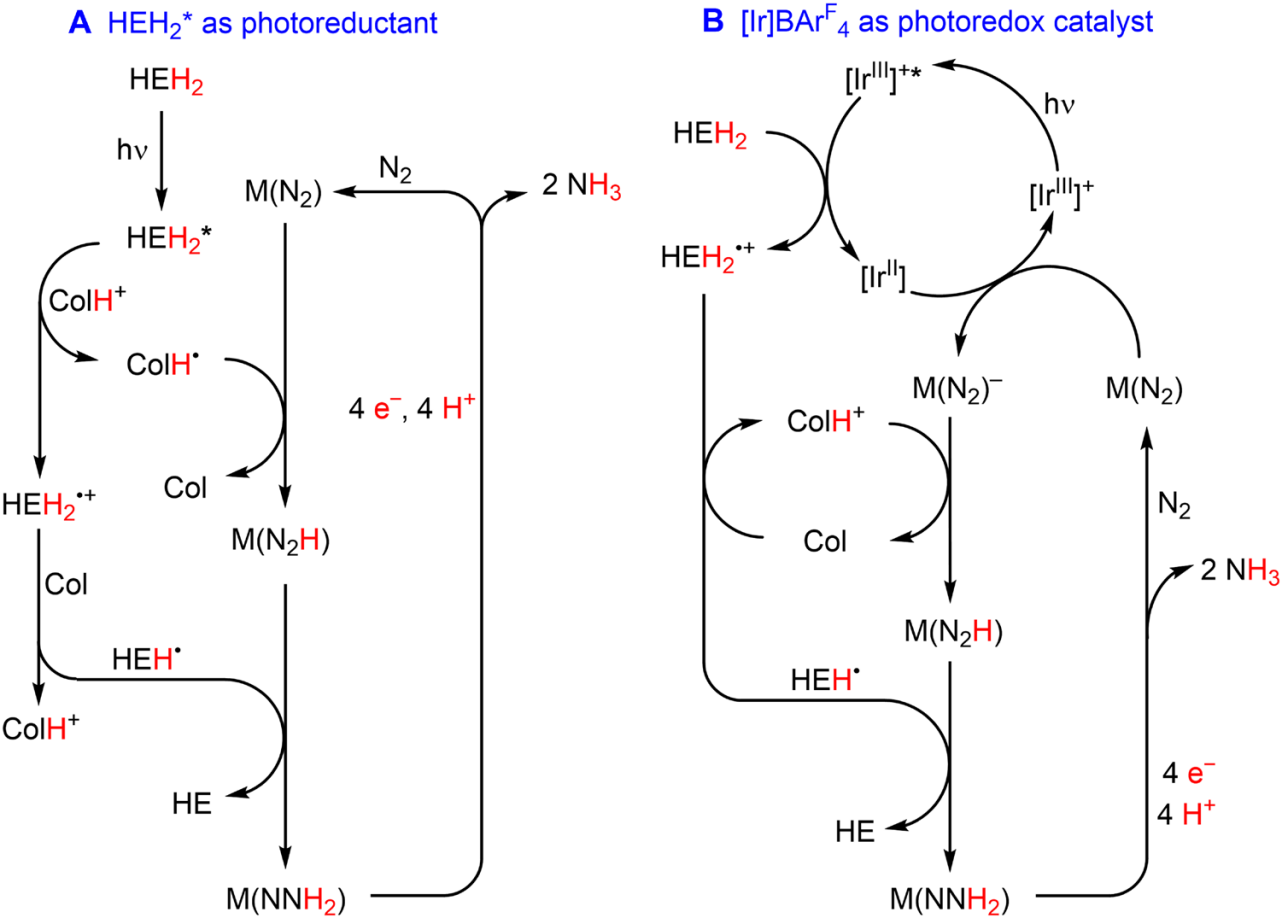
Possible scenarios for photodriven transfer hydrogenation from HEH_2_ to N_2_ mediated by a metal catalyst and buffer system (Col/[ColH]^+^). (**A**) Scenario in the absence of photoredox catalyst, in which [HEH_2_]* is oxidatively quenched by [ColH]^+^ to generate [ColH]^•^. (**B**) Scenario with photoredox catalyst, in which [Ir^III^]^+^* is reductively quenched by HEH_2_. Pathways involving N≡N bond cleavage to yield M≡N intermediates (not shown) are also plausible (fig. S21).

While future studies are needed to probe the mechanism of this transformation, the fate of photoexcited [HEH_2_]* is likely key. Two limiting scenarios to consider are the direct reduction of N_2_R intermediates by [HEH_2_]* (fig. S20) or the reduction of the [ColH]OTf to [ColH]^•^ radical, which then reacts with M(N_2_) (Fig. 3A) to form an N─H bond via M(N_2_H). Pyridinyl radicals have been posited as possible intermediates of N_2_R in thermally driven catalysis with molecular systems (*25*). Increasing the buffer concentration to 216 equiv per Mo boosted the NH_3_ yield to 20.3 ± 1.1 equiv of NH_3_/Mo (entry 8). This observation points to a pathway whereby reduction of [ColH]OTf by [HEH_2_]* dominates (Fig. 3A), consistent with the high reactivity expected of [HEH_2_]* (*E*_ox_ ~ −2.6 V; p*K*_a_ ~ −20; BDFE_C─H_ ~ −8.5 kcal mol^−1^) and its short solution lifetime [0.419 ns in dimethyl sulfoxide (DMSO) solvent at 25°C] (*18*, *20*). Accordingly, steady-state fluorimetry studies show efficient quenching of [HEH_2_]* upon titrating in [ColH]OTf (fig. S11). Similar titrations of Col revealed less-efficient quenching (fig. S12). However, as some NH_3_ can be detected under irradiation even in the absence of buffer (entry 5), other photoinduced pathways for N─H bond formation via HEH_2_ are accessible. The addition of 10 equiv of tetrabutylammonium bromide (TBABr) had no effect on the NH_3_ yield (entry 9), suggesting that reductive Br^−^ loss from the precatalyst is not a limiting factor.

Figure 3A provides a generalized mechanistic outline to help illustrate how a photon might facilitate delivery of H_2_ from HEH_2_ to M(N_2_), to first generate an M(NNH_2_) intermediate, and to ultimately generate NH_3_ via successive H_2_ transfers. For simplicity, we show only this one scenario in Fig. 3A but emphasize that other scenarios, including the early generation and then reduction of a terminal nitride intermediate (Mo≡N + HEH_2_ → Mo(NH_2_) + HE) (fig. S21), are also very plausible (*26*). A recent study showed that a Mn^V^≡N can be photoreduced by 9,10-dihydroacridine to liberate NH_3_ (*27*).

Limitations stemming from a short [HEH_2_]* excited-state lifetime and low-quantum yield (0.031) (*20*) for HEH_2_ motivated us to explore a photosensitizer to enhance this photodriven catalysis. To test this idea, [Ir(ppy)_2_(dtbbpy)]BAr^F^_4_ ([Ir]BAr^F^_4_; *E*_1/2_(Ir^III/II^) = −1.90 V) was chosen as its reduction potential is close to that of Cp*_2_Co and hence should be compatible with N_2_R using [Mo]Br_3_ (*21*, *28*).

Including [Ir]BAr^F^_4_ with [Mo]Br_3_ (1 equiv, both at 2.3 mM), in addition to 54 equiv each of HEH_2_ and Col/[ColH]OTf in THF, under an N_2_ atmosphere and blue LED irradiation for 12 hours at 23°C, yields 24 ± 4 equiv of NH_3_/Mo (entry 10). Assuming that HEH_2_ is a 2 e^−^/2 H^+^ donor, these conditions correspond to an overall NH_3_ yield of 67 ± 10% with respect to HEH_2_. Furthermore, in the presence of the Ir photosensitizer, catalytic amounts of buffer can be used, producing 15.8 ± 0.8 equiv of NH_3_/Mo (entry 11). In addition to higher yields, the inclusion of [Ir]BAr^F^_4_ enhances the photocatalytic rate; the catalysis is ~80% complete after 30 min (entry 12). By contrast, under Ir-free conditions, 2-hour reaction times are required to achieve ~80% completion (entry 13). Comparing this photodriven Mo-catalyzed N_2_R via HEH_2_ with thermally driven Mo-catalyzed N_2_R using (Cp*)_2_Co and [ColH]OTf as reported by Nishibayashi, we find that the NH_3_ yields with respect to reductant are quite similar (69% for the latter case) (*21*).

As in the Ir-free process, lowering the [Mo]Br_3_ loading increased the turnover for NH_3_ with catalytic buffer (26.0 ± 0.4 equiv of NH_3_/Mo; entry 14), but with decreased total yield. No NH_3_ is produced without irradiation (entry 15), and the presence of [Mo]Br_3_ and HEH_2_ are likewise essential (entries 16 and 17). Similar to the Ir-free reaction, HE was found to be the major organic product (>80%), and complete consumption of HEH_2_ was observed (fig. S4). Solvent screening suggests that the reaction is most efficient when all components are soluble (see table S5). By contrast, other catalytic N_2_R methods rely on low solubility of either the acid or the reductant to attenuate competing H_2_ evolution, demonstrating an advantage to using a terminal H atom source that is not competent for H_2_ release in the ground state (*1*).

A range of candidate H_2_ carriers, subH_2_, should be explored in future studies to identify donors whose spent products can be recycled efficiently, perhaps in situ, via hydrogenation with H_2_ or electrochemically (2 e^−^/2 H^+^). In an initial survey, the Ir-photosensitizer cocatalyst enables catalytic production of NH_3_ under irradiation with 9,10-dihydroacridine or 5,6-dihydrophenanthridine as the H_2_ donor (6.4 ± 0.3 equiv of NH_3_/Mo and 4.6 ± 0.8 equiv of NH_3_/Mo, respectively; entries 18 and 19). While noncatalytic, N_2_-to-NH_3_ conversion is also achieved with [Ir]BAr^F^_4_ and the H^−^ donor 1-benzyl-1,4-dihydronicotinamide (1.2 ± 0.1 equiv of NH_3_/Mo; entry 20). In the absence of [Ir]BAr^F^_4_, none of these H_2_ or H^−^ carriers are competent for the photoinduced N_2_RR (see table S2). The reaction with HEH_2_ tolerates a 1:1 mixture of N_2_ and H_2_ (1 atm of total pressure, 14 ± 4 equiv of NH_3_/Mo; entry 21), indicating that the Mo catalyst is not (at least irreversibly) poisoned by H_2_ under these conditions, important for considering downstream recycling of the spent donor.

In addition to varying the subH_2_, we have examined the effect of varying the Ir-photosensitizer. [Ir(dF(CF_3_)ppy)_2_(dtbbpy)]PF_6_ yielded substantially less NH_3_ (entry 22) than [Ir]PF_6_ (entry 23) or [Ir]BAr^F^_4_ (entries 10 and 12; Fig. 2). [Ir^II^(dF(CF_3_)ppy)_2_(dtbpy)] is also less reducing (*E*_1/2_(Ir^III/II^) = −1.75 V) (*29*), possibly pointing to a redox-based cutoff for photodriven N_2_R. Accordingly, [Ir(*p*-F(Me)ppy)_2_(dtbbpy)]PF_6_ (*E*_1/2_(Ir^III/II^) = −1.88 V) restores the yields observed in the parent system (entry 24) (*30*). However, Ir(ppy)_3_, despite having the strongest reduction potential (*E*_1/2_(Ir^III/II^) = −2.57 V), gave attenuated NH_3_ yields (entry 25) and therefore suggests that multiple factors may be at play.

Figure 3B provides a working model to account for the role of [Ir]BAr^F^_4_. Upon excitation of [Ir^III^]^+^ to [Ir^III^]^+^*, reductive quenching by HEH_2_ would generate [Ir^II^], as has been established in related reductions of organic substrates (Fig. 3B) (*9*). This proposed pathway is consistent with the lack of enhancement observed with Ir(ppy)_3_, with which reductive quenching by HEH_2_ is very uphill [*E*_1/2_(*Ir^III/II^) = −0.08 V, *E*_1/2_(HEH_2_^0/+^) = 0.48 V] (*29*). The resulting radical cation HEH_2_^•+^ is then deprotonated by Col, mitigating back-electron transfer from [Ir^II^]. As noted above, [Ir^II^] is assumed to be sufficiently reducing to generate an M(N_2_)^−^ species from M(N_2_). The former would then undergo protonation by [ColH]^+^ to form an N─H bond via M(N_2_H), which itself can be reduced further by diffusing HEH^•^ to generate M(NNH_2_). As noted for Fig. 3A, this series of steps is plausible but is only one of several related scenarios that may be viable (e.g., [Ir^II^] might be oxidized by [ColH]^+^ instead of a [Mo] species), and future mechanistic studies are needed.

In contrast to the Ir-free conditions, the system with the photosensitizer remains catalytically competent even without added buffer, albeit with an attenuation in turnover (7.4 ± 0.4 equiv of NH_3_/Mo; entry 26). Presumably, under a Col/[ColH]^+^-free cycle, the liberated radical cation HEH_2_^•+^ (formed via reductive quenching) can be consumed via proton or H atom transfer with a [Mo]N*_x_*H*_y_* intermediate.

Having established photodriven transfer hydrogenation as a viable strategy for N_2_R, we have begun to explore the deep reduction of other substrates. While success here will ultimately be best realized by exploring a broader array of transition metal catalysts, promising early results with the [Mo]Br_3_ catalyst discussed here include the complete reduction of nitrate to ammonia (8 e^−^/9 H^+^) and acetylene to ethylene (major product; 2 e^−^/2 H^+^) and ethane (minor product; 4 e^−^/4 H^+^). These transformations have been previously explored by photochemical methods, including with semiconductors as for N_2_ (*31*, *32*). Also of relevance is the photoinduced hydroalkylation of alkynes using Hantzsch ester derivatives, although transfer hydrogenation from HEH_2_ to acetylene has not, to our knowledge, been previously reported (*33*).

Reduction of [TBA]NO_3_ with HEH_2_ in the presence of buffer and [Mo]Br_3_ under blue LED irradiation and argon atmosphere yields 9.8 ± 1.2 equiv of NH_3_/Mo, representing a 73 ± 9% yield with respect to HEH_2_ (Fig. 2, entry 27). The reaction carried out with [TBA]^15^NO_3_ yielded ^15^NH_3_ (fig. S16), confirming NO_3_^−^ as the source of N atoms. In contrast to N_2_R, addition of [Ir]BAr^F^_4_ did not enhance catalysis (entry 28). Distinct from N_2_ as the substrate where no background reactivity is observed (entry 3), there is some background reactivity for NO_3_^−^ even in the absence of the Mo catalyst; this reactivity is enhanced by the Ir photocatalyst (entries 29 and 30; see section S5.5 for further discussion). Only trace NH_3_ was detected in the absence of light (entry 31).

The reduction of acetylene under the same conditions (HEH_2_, Col/[ColH]OTf buffer, and [Mo]Br_3_ under blue LED irradiation and argon atmosphere) provides a mixture of ethylene and ethane in a ~6:1 ratio and a total yield of 24 ± 5% with respect to HEH_2_ (entry 32). Addition of [Ir]BAr^F^_4_ to this reaction marginally decreases the yield (entry 33). However, as in the NO_3_^−^ reduction reaction, [Ir]BAr^F^_4_ enhances Mo-free reactivity (entries 34 and 35). Again, no reduced products could be detected in the absence of light (entry 36). In sum, each of these three substrates (N_2_, NO_3_^−^, and HCCH) illustrates the capacity of HEH_2_ to deliver H_2_ equivalents via photodriven transfer hydrogenation.

**Fig. 4.**
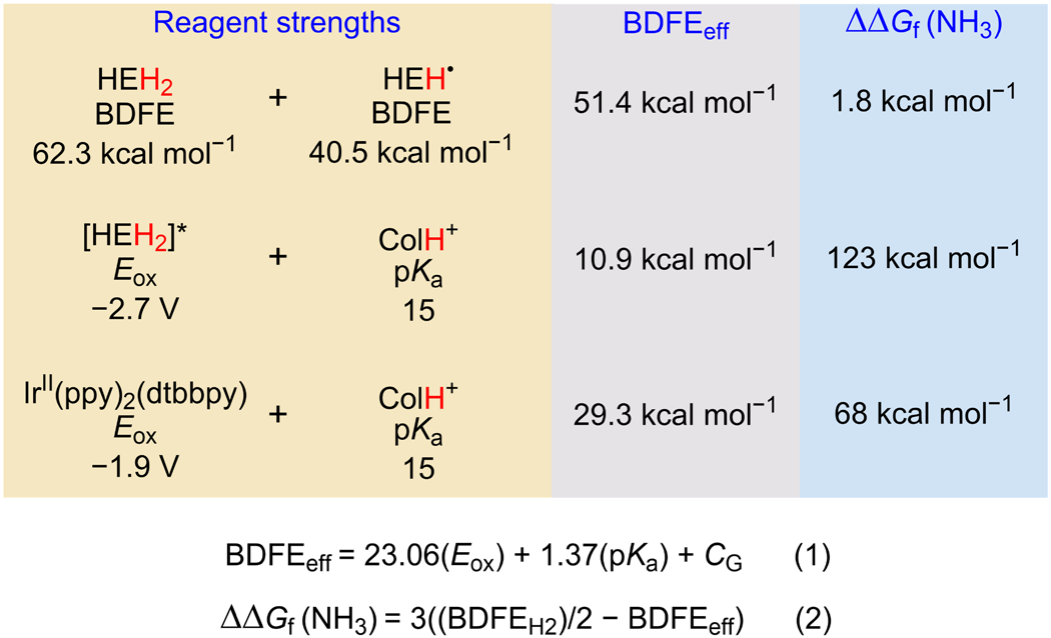
Estimated BDFE_eff_ values and corresponding ΔΔ*G*_f_(NH_3_) for the transformations of interest here. Values are estimated using Eqs. 1 and 2.

To close, it is instructive to consider the thermodynamics of the photodriven N_2_R system described here and its hypothetical dark reaction (Fig. 1C). To do this, one can compare the BDFE_eff_ (Fig. 4, Eq. 1), a measure of the thermodynamics of H atom transfer from a set of reagents, to the BDFE of H_2_ (103.9 kcal mol^−1^) (*34*–*36*). The difference between these values provides an overpotential for N_2_ hydrogenation, expressed as ΔΔ*G*_f_(NH_3_) (Eq. 2) (*37*). For the dark reaction, the BDFE_eff_ is the average of the first (C─H) and second (N─H) BDFEs for HEH_2_ and HEH^•^, respectively, correlating to a very small overpotential [ΔΔ*G*_f_(NH_3_) = 1.8 kcal mol^−1^] (*18*). NH_3_ synthesis via transfer hydrogenation from HEH_2_ to N_2_ is therefore thermodynamically comparable to N_2_ hydrogenation by the Haber-Bosch process. Where the latter uses high temperature and pressure to overcome the high kinetic barrier, the photodriven process described here obtains excess driving force directly from visible light. More specifically, under conditions that exclude the photosensitizer, using the estimated excited-state reduction potential of [HEH_2_]* and the p*K*_a_ of [ColH]^+^ to estimate BDFE_eff_, blue light affords access to a large added driving force [ΔΔ*G*_f_(NH_3_) = 123 kcal mol^−1^; Fig. 4] to push the transfer hydrogenation forward. In the presence of the Ir photosensitizer, a smaller but still considerable driving force [ΔΔ*G*_f_(NH_3_) = 68 kcal mol^−1^] is available. Regardless, the key point is that light generates an overpotential from an otherwise unreactive source of 2 e^−^/2 H^+^ stored within HEH_2_ that is sufficient to perform, via successive transfers, a net 6 e^−^/6 H^+^ reduction of N_2_ in the presence of an appropriate catalyst and cocatalyst buffer, with an additional benefit gained from inclusion of a photoredox cocatalyst. Important future goals for the work presented here include extensive mechanistic studies and studies aimed at in situ recycling of the spent HE back to HEH_2_.

## MATERIALS AND METHODS

### Experimental design

To develop and study photodriven N_2_R, catalytic reactions were performed, and their fixed-N products were quantified using a variety of reagents, (co)catalysts, and conditions. Additional spectroscopic experiments were conducted to gain mechanistic insight.

### General considerations

All manipulations were carried out using standard Schlenk or glovebox techniques under an N_2_ atmosphere. Solvents were deoxygenated and dried by thoroughly sparging with N_2_ followed by passage through an activated alumina column in a solvent purification system by SG Water USA LLC. Nonhalogenated solvents were tested with sodium benzophenone ketyl in THF to confirm the absence of oxygen and water. Deuterated solvents were purchased from Cambridge Isotope Laboratories Inc., degassed, and dried over activated 3-Å molecular sieves before use.

### Reagents

HEH_2_ (*38*), (PNP)MoBr_3_ (*21*), [ColH]OTf (*21*), [P_3_^B^Fe]BAr^F^_4_ [P_3_^B^ (tris[2-(diisopropylphosphino)phenyl]borane)] (*39*), BTH_2_ (*18*), NaBAr^F^_4_ (*40*), ^15^N-Col (*41*), phenH_2_ (*42*), phenazH_2_ (*43*), and [TBA]^15^NO_3_ (*44*) were prepared according to literature procedures. Triflic acid, ethylacetoacetate, and 37% aqueous formaldehyde were purchased from Sigma-Aldrich and used without further purification. Ir(ppy)_3_, [Ir(ppy)_2_(dtbbpy)]PF_6_, [Ir(dF(CF_3_)ppy)_2_(dtbbpy)]PF_6_, and [Ir(*p*-F(Me)ppy)_2_(dtbbpy)]PF_6_ were purchased from Strem and used without further purification. [TBA]NO_3_ was purchased from Alfa Aesar and was dissolved in THF and filtered over activated alumina to dry and purify before use. ^15^N_2_ was obtained from Cambridge Isotope Laboratories Inc. (lot number: I-25854/XZ732957). ^15^NH_4_Cl (99% ^15^N, 98% purity) and Na^15^NO_3_ (98% ^15^N, 98% purity) was purchased from Cambridge Isotope Laboratories Inc. and used without further purification. Col was purchased from Sigma-Aldrich and was distilled before use. 9,10-Dihydroacridine (98%) was purchased from Combi Blocks and used without further purification. 1-benzyl-1,4-dihydronicotinamide was purchased from TCI and used without further purification. Acetylene (99.6% purity) was purchased from Matheson Gas. THF used in the experiments here was stirred over Na/K (≥12 hours) and filtered over activated alumina or vacuum-transferred before use unless otherwise stated. Photoinduced reactions were performed using Kessil 34-W 150 blue lamps.

### Spectroscopy

#### Nuclear magnetic resonance spectroscopy

Nuclear magnetic resonance (NMR) measurements were recorded with a Varian 400-MHz spectrometer. ^1^H NMR chemical shifts are reported in parts per million (ppm) relative to tetramethylsilane, using ^1^H resonances from residual solvent as internal standards (*45*).

#### Ultraviolet-visible spectroscopy

Ultraviolet-visible (UV-vis) absorption spectroscopy measurements were collected with a Cary 50 UV-vis spectrophotometer using a 1-cm path length quartz cuvette. All samples had a blank sample background subtraction applied.

#### Electron paramagnetic resonance spectroscopy

All X-band continuous-wave electron paramagnetic resonance spectra were obtained on a Bruker EMX spectrometer using a quartz liquid nitrogen immersion dewar on solutions prepared as frozen glasses in 2-MeTHF, unless otherwise noted.

#### Steady-state fluorimetry

Steady-state fluorimetry was performed in the Beckman Institute Laser Resource Center (California Institute of Technology). Samples for luminescence measurements were prepared in dry THF and transferred to a 1-cm path length–fused quartz cuvette sealed with a high-vacuum Teflon valve (Kontes). Steady-state emission spectra were collected on the Jobin S4 Yvon Spec Fluorolog-3-11 with a Hamamatsu R928P photomultiplier tube detector with photon counting.

### Standard NH_3_ generation reaction procedure

All solvents are stirred with Na/K for ≥2 hours and filtered before use. In a nitrogen-filled glovebox, the precatalysts ([Mo]Br_3_ and/or [Ir]BAr^F^_4_) (2.3 μmol) are weighed in individual vials. The precatalysts are then transferred quantitatively into a Schlenk tube using THF, and the THF is evaporated to provide a thin film of precatalyst. The tube is then charged with a stir bar, and the acid ([ColH]OTf) and Hantzsch ester (HEH_2_) are added. The tube is cooled to 77 K in a cold well. The base (Col) is dissolved in 1 ml of solvent. The 1-ml solution of base and solvent is added to the cold tube to produce a concentration of the precatalyst of 2.3 mM. The temperature of the system is allowed to equilibrate for 5 min, and then the tube is sealed with a Teflon screw valve. The tube is passed out of the box into a liquid N_2_ bath and transported to a fume hood. For experiments run at −78°C, the tube is then transferred to a dry ice/isopropanol bath where it thaws and is allowed to stir under blue LED irradiation for a minimum of 3 hours before warming. For experiments run at 23°C, the tube is instead transferred to a water bath where it thaws and is allowed to stir for 12 hours. To ensure reproducibility, all experiments were conducted in 200-ml Schlenk tubes (50 mm outer diameter) using 10-mm egg-shaped stir bars, and stirring was conducted at ~600 rpm. Both the water bath and the dry ice/isopropanol bath were contained in highly reflective dewars. The blue LED was placed above the bath as close to the stirring reaction as possible.

### NH_3_ detection by optical methods

Reaction mixtures are cooled to 77 K and allowed to freeze. The reaction vessel is then opened to atmosphere, and excess of a solution of HCl (3 ml of a 2.0 M solution in Et_2_O; 6 mmol) is slowly added to the frozen solution over 1 to 2 min. This solution is allowed to freeze, and then the headspace of the tube is evacuated and the tube is sealed. The tube is then allowed to warm to room temperature (RT) and stirred at RT for at least 10 min. Solvent is removed in vacuo, and the solids are extracted with 1 M HCl(aq) and filtered to give a total solution volume of 10 ml. A 5-ml aliquot is taken and washed repeatedly with *n*-butanol to remove Hantzsch pyridine (HE) and [ColH]^+^. After *n*-butanol washing, additional 1 M HCl(aq) is added to give a final total volume of 5 ml. From these 5-ml solutions, a 100-μl aliquot is analyzed for the presence of NH_3_ (present as [NH_4_]Cl) by the indophenol method. Quantification was performed with UV-vis spectroscopy by analyzing the absorbance at 635 nm (*46*). When specified, a further aliquot of this solution was analyzed for the presence of N_2_H_4_ (present as [N_2_H_5_]Cl) by a standard colorimetric method (*47*). Quantification was performed with UV-vis spectroscopy by analyzing the absorbance at 458 nm.

### NH_3_ detection by ^1^H NMR spectroscopy

Reaction mixtures are cooled to 77 K and allowed to freeze. The reaction vessel is then opened to atmosphere, and an excess (with respect to acid) solution of a NaO^t^Bu solution in MeOH (0.25 mM) is slowly added to the frozen solution over 1 to 2 min. This solution is allowed to freeze, and then the headspace of the tube is evacuated and the tube is sealed. The tube is then allowed to warm to RT and stirred at RT for at least 10 min. An additional Schlenk tube is charged with HCl (3 ml of a 2.0 M solution in Et_2_O; 6 mmol) to serve as a collection flask. The volatiles of the reaction mixture are vacuum-transferred at RT into this collection flask. After completion of the vacuum transfer, the collection flask is sealed and warmed to RT. Solvent is removed in vacuo, and the remaining residue is dissolved in 0.7 ml of DMSO-*d*_6_ containing 20 mM 1,3,5-trimethoxybenzene as an internal standard. Integration of the ^1^H NMR peak observed for NH_4_^+^ is then integrated against the two peaks of trimethoxybenzene to quantify the ammonium present. This ^1^H NMR detection method was also used to differentiate [^14^NH_4_]Cl and [^15^NH_4_]Cl produced in the control reactions conducted with ^15^N_2_, ^15^N-Col/[ColH]OTf, or ^15^N-HEH_2_.

### Standard [TBA]NO_3_ reduction reaction procedure

Catalytic experiments for the reduction of [TBA]NO_3_ were conducted in a manner similar to the reduction of N_2_. The precatalysts, solids, and stir bar are added in the same way, with [TBA]NO_3_ included with the other solids. The tube is cooled to 77 K in a cold well, and the base (Col) is added using a micropipette. The tube is then sealed and passed out of the glovebox without warming and thoroughly degassed. Degassed THF solvent (1 ml) is vacuum-transferred into the catalytic tube. The tube is allowed to warm briefly and back-filled with argon. The reaction is then irradiated with blue LED in a 23°C water bath as for the N_2_RR.

### Standard acetylene reduction reaction procedure

Catalytic experiments for the reduction of acetylene were conducted in a manner similar to the reduction of N_2_. The precatalysts, solids, and stir bar are added in the same way. The tube is wrapped in aluminum foil, and Col and THF-*d*_8_ (0.7 ml) are added. The tube is sealed, passed out of the glovebox, and degassed (three freeze-pump thaw cycles). The desired volume of acetylene gas is added using a calibrated bulb while the tube is cooled in liquid nitrogen. The headspace of the tube is then backfilled to 1 atm with argon while cooled in a dry ice/acetone bath. The tube is transferred to a 23°C water bath and is irradiated with blue LED for the time specified.

After 12 hours of irradiation, the volatiles of the reaction mixture are vacuum-transferred into a J. Young NMR tube of known volume containing a known amount of 1,3,5-trimethoxybenzene. In the ^1^H NMR spectrum of the resulting sample, the peaks corresponding to ethylene (5.36 ppm) and ethane (0.85 ppm) are distinguishable when present (*45*). Integration to the internal standard provides the yield of dissolved gases. Henry’s constant for each gas in THF (*48*) was used to estimate their partial pressures in the headspace.

### Synthetic details

#### ^15^N-labeled 2,6-dimethyl-3,5-dicarboethoxy-l,4-dihydropyridine (^15^N-HEH_2_)

Adapted from (*38*), aqueous formaldehyde (37%, 78 μl) and ethylacetoacetate (280 μl, 2.19 mmol) were placed in a 10-ml round-bottom flask equipped with a stir bar and fitted with a reflux condenser. ^15^NH_4_Cl (305 mg, 5.7 mmol) in 1 ml of H_2_O was added to a 1-ml aqueous solution of NaOH (228.3 mg, 5.7 mmol). The resulting solution of ^15^NH_4_OH was added to the flask through the neck of the condenser. The condenser neck was rinsed into the flask with 0.5 ml of ethanol. The reaction mixture was heated at reflux for 1.5 hours and then chilled in an ice bath. The resulting precipitate was collected by filtration and washed with cold ethanol (~3 ml) and Et_2_O to yield the title compound as a pale yellow powder (60 mg, 22% yield). ^1^H NMR (400 MHz, DMSO-*d*_6_) δ 8.28 ppm (d, ^1^*J*_H,N_ = 94.6 Hz, 1H), 4.05 ppm (q, *J* = 7.1 Hz, 4H), 3.11 ppm (s, 2H), 2.11 ppm (d, *J* = 2.9 Hz, 6H), and 1.19 ppm (t, *J* = 7.1 Hz, 6H).

#### ^15^N-labeled 2,4,6-trimethylpyridinium triflate (^15^N-[ColH]OTf)

Identical procedure to what has previously been reported with unlabeled Col was used (*21*). ^1^H NMR (400 MHz, DMSO*-d*_6_) δ 14.87 ppm (broad s, 1H), 7.57 ppm (d, ^3^*J*_H,N_ = 2.8 Hz, 2H), 2.62 ppm (d, ^3^*J*_H,N_ = 2.9 Hz, 6H), and 2.49 ppm (s, 3H).

#### [Ir(ppy)_2_(dtbbpy)]BAr^F^_4_ ([Ir]BAr^F^_4_)

[Ir(ppy)_2_(dtbbpy)]PF_6_ (100 mg, 0.11 mmol) and NaBAr^F^_4_ (92.2 mg, 0.10 mmol, 0.95 equiv) were stirred in 5 ml of Et_2_O at RT for 1 hour. The solution was filtered through celite, layered with pentane, and stored at −40°C overnight to yield the title compound as yellow crystals (161 mg, 90% yield). ^1^H NMR (400 MHz, MeCN-*d*_3_) δ 8.48 ppm (s, 2H), 8.06 ppm (d, 2H, *J* = 8.2 Hz), 7.93 to 7.76 ppm (m, 6H), 7.74 to 7.64 ppm (m, 10H), 7.58 ppm (d, *J* = 5.8 Hz, 2H), 7.50 ppm (dd, *J* = 5.9, 1.9 Hz, 2H), 7.03 ppm (t, *J* = 6.8 Hz, 2H), 6.91 ppm (t, *J* = 6.8 Hz, 2H), 6.28 ppm (d, *J* = 6.3 Hz, 2H), and 1.40 ppm (s, 18H).

## Supplementary Materials

### This PDF file includes:

Sections S1 to S9 Figs. S1 to S24 Tables S1 to S11 References
