# Supplementary material for: Catalytic transfer hydrogenation of N2 to NH3 via a photoredox catalysis strategy
Source: Sci Adv. 2022 Oct 26;8(43):eade3510. doi: 10.1126/sciadv.ade3510 (PMC9604530; doi:10.1126/sciadv.ade3510)
Supplement: Supplementary file 1 — Sections S1 to S9 Figs. S1 to S24 Tables S1 to S11 References [file sciadv.ade3510_sm.pdf]

Science Advances
AAAS

Supplementary Materials for

Catalytic transfer hydrogenation of N_2 to NH_3 via a photoredox catalysis strategy

Christian M. Johansen, Emily A. Boyd, Jonas C. Peters

Corresponding author: Jonas C. Peters, jpeters@caltech.edu

Sci. Adv. **8**, eade3510 (2022)
DOI: 10.1126/sciadv.ade3510

This PDF file includes:

Sections S1 to S9
Figs. S1 to S24
Tables S1 to S11
References

S1 Ammonia production and quantification studies

The ammonia generation procedures are also described in the **Methods and Materials** section but are redescribed with some additional detail here for the benefit of the reader.

S1.1 Standard NH_3 Generation Reaction Procedure

All solvents are stirred with Na/K for ≥2 hours and filtered prior to use. In a nitrogen-filled glovebox, the precatalysts ([Mo]Br_3 and/or [Ir]BAr^F_4) (2.3 µmol) are weighed in individual vials.* The precatalysts are then transferred quantitatively into a Schlenk tube using THF. The THF is then evaporated to provide a thin film of precatalyst at the bottom of the Schlenk tube. The tube is then charged with a stir bar and the acid and Hantzsch ester (HEH_2) are added as solids. The tube is cooled to 77 K in a cold well. The base ([Col]) is dissolved in 1 mL solvent. To the cold tube is added the 1 mL solution of base and solvent to produce a concentration of precatalyst of 2.3 mM. The temperature of the system is allowed to equilibrate for 5 minutes and then the tube is sealed with a Teflon screw-valve. This tube is passed out of the box into a liquid N_2 bath and transported to a fume hood. For experiments run at -78 °C the tube is then transferred to a dry ice/isopropanol bath where it thaws and is allowed to stir under blue LED irradiation at -78 °C for minimum three hours before warming. For experiments run at 23 °C the tube is instead transferred to a water bath where it thaws and is allowed to stir for 12 hours. To ensure reproducibility, all experiments were conducted in 200 mL Schlenk tubes (50 mm OD) using 10 mm eggshaped-stir bars and stirring was conducted at ~600 rpm. Both the water bath and the dry ice/isopropanol bath were contained in highly reflective dewars. The Blue LED was placed above the bath as close to the stirring reaction as possible.

* In cases where less than 2.3 µmol of precatalyst were used, stock solutions were used to avoid having to weigh very small amounts.

S1.2 NH_3 Generation Reaction Procedure under Partial H_2 Atmosphere

Catalytic runs done under a mixture of H_2 and N_2 were conducted similarly to those under N_2 atmosphere, with a few differences described below. The loadings were the same as in Figure 2, Entry 10.

Catalysis is performed in the same Schlenk tubes as under N_2, which are charged with precatalyst, HEH_2, [ColH]OTf, and a stirbar in a nitrogen-filled glovebox as described above. After addition of the solids, the tube is wrapped in aluminum foil and the base (Col) is added in 1 mL of Na/K dried THF at room temperature. Half of the headspace volume is then removed using a calibrated bulb and then backfilled with H_2 which has been passed through a liquid nitrogen trap. The aluminum foil is removed and the reaction is allowed to stir under Blue LED irradiation for 12 hours. Variation from the standard procedure (addition of THF/Col at room temperature and allowing to stir without irradiation for 30 min before exposing to blue LED) were found to not perturb the yield of NH_3.

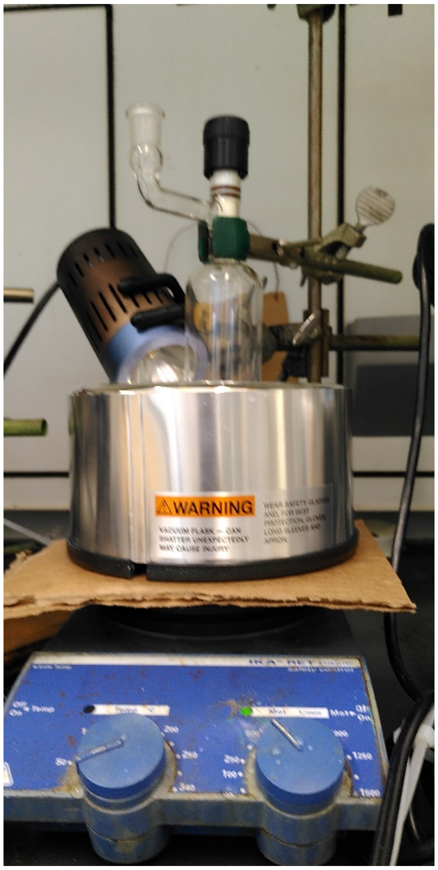

Figure S1. Set-up for catalysis with Schlenk tube, stir plate, Kessil® 34 W 150 Blue lamp and dewar. Lamp is turned off for clarity.

S1.3 NH_3 detection by optical methods

Reaction mixtures are cooled to 77 K and allowed to freeze. The reaction vessel is then opened to atmosphere and to the frozen solution is slowly added excess of a solution of HCl (3 mL of a 2.0 M solution in Et_2O, 6 mmol) over 1-2 minutes. This solution is allowed to freeze, then the headspace of the tube is evacuated and the tube is sealed. The tube is then allowed to warm to RT and stirred at RT for at least 10 minutes. Solvent is removed *in vacuo*, and the solids are extracted with 1 M HCl(aq) and filtered to give a total solution volume of 10 mL. A 5 mL aliquot is taken and washed repeatedly with n-butanol to remove Hantzsch pyridine (HE) and collidinium. After n-butanol washing additional 1 M HCl(aq) is added to give a final total volume of 5 mL. From these 5 mL solutions, a 100 µL aliquot is analyzed for the presence of NH_3 (present as $[NH_4][Cl]$) by the indophenol method. Quantification was performed with UV-vis spectroscopy by analyzing the absorbance at 635 nm.(*46*) When specified a further aliquot of this solution was analyzed for the presence of N_2H_4 (present as $[N_2H_5][Cl]$) by a standard colorimetric method.(*47*) Quantification was performed with UV-vis spectroscopy by analyzing the absorbance at 458 nm.

S1.4 NH_3 detection by ^{1}H NMR

Reaction mixtures are cooled to 77 K and allowed to freeze. The reaction vessel is then opened to atmosphere and to the frozen solution is slowly added an excess (with respect to acid) solution of a

NaO^tBu solution in MeOH (0.25 mM) over 1-2 minutes. This solution is allowed to freeze, then the headspace of the tube is evacuated and the tube is sealed. The tube is then allowed to warm to RT and stirred at RT for at least 10 minutes. An additional Schlenk tube is charged with HCl (3 mL of a 2.0 M solution in Et_2O, 6 mmol) to serve as a collection flask. The volatiles of the reaction mixture are vacuum transferred at RT into this collection flask. After completion of the vacuum transfer, the collection flask is sealed and warmed to RT. Solvent is removed in vacuo, and the remaining residue is dissolved in 0.7 mL of DMSO-d_6 containing 20 mM 1,3,5-trimethoxybenzene as an internal standard. Integration of the ^1H NMR peak observed for NH_4^+ is then integrated against the two peaks of trimethoxybenzene to quantify the ammonium present. This ^1H NMR detection method was also used to differentiate [$^{14}NH_4$][Cl] and [$^{15}NH_4$][Cl] produced in the control reactions conducted with $^{15}N_2$, ^{15}N-Col/[ColH]OTf, or ^{15}N-HEH_2.

S1.5 NH_3 detection results

S1.5.1 Catalytic results in main text (Figure 2)

Table S1: Catalytic yields for photodriven transfer hydrogenation of N_2 to NH_3.

$$N_2 + 3\,HEH_2 \xrightarrow[\text{THF, 23 °C; 12 hrs; Blue LED}]{PNPMoBr_3;\ \text{Col/[ColH]OTf}} 2\,NH_3 + 3\,HE$$

Run	Conditions	[Mo] load (μmol)	acid (μmol)	base (μmol)	Ir (μmol)	HEH_2 equiv /Mo	NH_3 equiv /Mo	N_2H_4 equiv/ Mo	NH_3 yield/ HEH_2 (%)
**Figure 2, entry 1: Standard conditions**									
**A1**	THF, 23 °C	2.3	124.2	124.2	0	54	9.5	-	
**B1**	THF, 23 °C	2.3	124.2	124.2	0	54	8.3	-	
**C1**	THF, 23 °C	2.3	124.2	124.2	0	54	10.8	-	
	THF, 23 °C	2.3	124.2	124.2	0	54	9.5±1		26.5±3
**Figure 2, entry 2: 0.575 mM [Mo]Br_3**									
**D1**	THF, 23 °C	0.575	124.2	124.2	0	216	22.6	-	
**E1**	THF, 23 °C	0.575	124.2	124.2	0	216	20.9	-	
	THF, 23 °C	0.575	124.2	124.2	0	216	21.8±0.8		15.1±06
**Figure 2, entry 3: No Mo**									
**F1**	THF, 23 °C	0	124.2	124.2	0	54	<0.1	<0.1	
**G1**	THF, 23 °C	0	124.2	124.2	0	54	<0.1	<0.1	
	THF, 23 °C	2.3	124.2	124.2	0	54	<0.1	<0.1	<0.3
**Figure 2, entry 4: No light**									
**H1**	THF, 23 °C no light	2.3	124.2	124.2	0	54	<0.1	<0.1	
**I1**	THF, 23 °C no light	2.3	124.2	124.2	0	54	<0.1	<0.1	
	THF, 23 °C no light	2.3	124.2	124.2	0	54	<0.1	<0.1	<0.3
**Figure 2, entry 5: No buffer**									
**J1**	THF, 23 °C	2.3	**0**	**0**	0	54	0.74	-	
**K1**	THF, 23 °C	2.3	**0**	**0**	0	54	1.11	-	
	THF, 23 °C	2.3	**0**	**0**	0	54	0.9±0.2		2.6±0.5
**Figure 2, entry 6: 5 equiv Col/[ColH]OTf**									
**L1**	THF, 23 °C	2.3	11.5	11.5	0	54	2.7	<0.1	
**M1**	THF, 23 °C	2.3	11.5	11.5	0	54	3.2	<0.1	
**N1**	THF, 23 °C	2.3	11.5	11.5	0	54	2.8	-	
	THF, 23 °C	2.3	11.5	11.5	0	54	2.9±0.2	<0.1	8.1±0.6
**Figure 2, entry 7: benzene instead of THF**									
**O1**	C_6H_6, 23 °C	2.3	124.2	124.2	0	54	4.8	-	
**P1**	C_6H_6, 23 °C	2.3	124.2	124.2	0	54	4.6	-	
	C_6H_6, 23 °C	2.3	124.2	124.2	0	54	4.7±0.1		13±0.3

Run	Conditions	[Mo] load (µmol)	acid (µmol)	base (µmol)	Ir (µmol)	HEH_2 equiv /Mo	NH_3 equiv /Mo	N_2H_4 equiv /Mo	NH_3 yield/ HEH_2 (%)
**Figure 2, entry 8: 216 equiv Col/[ColH]OTf**									
**Q1**	THF, 23 °C	2.3	496.8	496.8	0	54	19.5	-	
**R1**	THF, 23 °C	2.3	496.8	496.8	0	54	21.1	-	
	THF, 23 °C	2.3	496.8	496.8	0	54	20.3±0.8		56±2
**Figure 2, entry 9: with 10 equiv TBABr**									
**S1**	THF, 23 °C	2.3	124.2	124.2	0	54	9	-	
**T1**	THF, 23 °C	2.3	124.2	124.2	0	54	8.6	-	
	THF, 23 °C	2.3	124.2	124.2	0	54	8.8±0.3		23.6±0.8
**Figure 2, entry 10: Added [Ir]BAr^F_4**									
**U1**	THF, 23 °C	2.3	124.2	124.2	2.3	54	29.8	-	
**V1**	THF, 23 °C	2.3	124.2	124.2	2.3	54	20.6	-	
**W1**	THF, 23 °C	2.3	124.2	124.2	2.3	54	20.5	-	
**X1**	THF, 23 °C	2.3	124.2	124.2	2.3	54	25.4	-	
	THF, 23 °C	2.3	124.2	124.2	2.3	54	24±4		67±10
**Figure 2, entry 11: Added [Ir]BAr^F_4, 5 equiv Col/[ColH]OTf**									
**Y1**	THF, 23 °C	2.3	11.5	11.5	2.3	54	16.02	<0.1	
**Z1**	THF, 23 °C	2.3	11.5	11.5	2.3	54	16.6	<0.1	
**AA1**	THF, 23 °C	2.3	11.5	11.5	2.3	54	14.7		
	THF, 23 °C	2.3	11.5	11.5	2.3	54	15.8±0.8	<0.1	44±2
**Figure 2, entry 12: Added [Ir]BAr^F_4, t = ½ h**									
**AB1**	THF, 23 °C t = ½ h	2.3	124.2	124.2	2.3	54	19.5	-	
**AC1**	THF, 23 °C t = ½ h	2.3	124.2	124.2	2.3	54	17.7	-	
	THF, 23 °C t = 1/2 h	2.3	124.2	124.2	2.3	54	18.6±0.9		52±3
~75 % completion compared to entry 10									
**Figure 2, entry 13: t = 2 h**									
**AD1**	THF, 23 °C t = 2 h	2.3	124.2	124.2	0	54	4.9	-	
**AE1**	THF, 23 °C t = 2 h	2.3	124.2	124.2	0	54	7.9	-	
**AF1**	THF, 23 °C t = 2 h	2.3	124.2	124.2	0	54	10	-	
	THF, 23 °C t = 2 h	2.3	124.2	124.2	0	54	7.6±2		21±6
~80 % completion compared to entry 1									
**Figure 2, entry 14: Added [Ir]BAr^F_4, 5 equiv Col/[ColH]OTf, 0.575 mM [Mo]Br_3**									
**AG1**	THF, 23 °C	0.575	11.5	11.5	2.3	216	26.83	-	
**AH1**	THF, 23 °C	0.575	11.5	11.5	2.3	216	25.96	-	
	THF, 23 °C	0.575	11.5	11.5	2.3	216	26±0.4		18.4±0.4
**Figure 2, entry 15: Added [Ir]BAr^F_4, 5 equiv Col/[ColH]OTf, no light**									
**AI1**	THF, 23 °C no light	2.3	11.5	11.5	2.3	54	<0.1	<0.1	
**AJ1**	THF, 23 °C no light	2.3	11.5	11.5	2.3	54	<0.1	<0.1	
	THF, 23 °C no light	2.3	11.5	11.5	2.3	54	<0.1	<0.1	<0.3

Run	Conditions	[Mo] load (µmol)	acid (µmol)	base (µmol)	Ir (µmol)	HEH_2 equiv /Mo	NH_3 equiv /Mo	N_2H_4 equiv/ Mo	NH_3 yield/ HEH_2 (%)
**Figure 2, entry 16: Added [Ir]BAr$^F_4$, 5 equiv Col/[ColH]OTf, no [Mo]Br_3**									
**AK1**	THF, 23 °C	**0**	11.5	11.5	2.3	54	<0.1	<0.1	
**AL1**	THF, 23 °C	**0**	11.5	11.5	2.3	54	<0.1	<0.1	
	THF, 23 °C	**0**	11.5	11.5	2.3	54	<0.1	<0.1	<0.3
**Figure 2, entry 17: Added [Ir]BAr$^F_4$, 5 equiv Col/[ColH]OTf, no HEH_2**									
**AM1**	THF, 23 °C	2.3	11.5	11.5	2.3	**0**	<0.1	<0.1	
**AN1**	THF, 23 °C	2.3	11.5	11.5	2.3	**0**	<0.1	<0.1	
	THF, 23 °C	2.3	11.5	11.5	2.3	**0**	<0.1	<0.1	<0.3
**Figure 2, entry 18: Added [Ir]BAr$^F_4$, subH_2 = 9,10-dihydroacridine**									
**AO1**	THF, 23 °C	2.3	124.2	124.2	2.3	54[a]	6.7	-	
**AP1**	THF, 23 °C	2.3	124.2	124.2	2.3	54[a]	6.1	-	
	THF, 23 °C	2.3	124.2	124.2	2.3	54[a]	6.4±0.3		17.7±0.8
[a]9,10-dihydroacridine used instead of HEH_2									
**Figure 2, entry 19: Added [Ir]BAr$^F_4$, subH_2 = 5,6-dihydrophenanthridine**									
**AQ1**	THF, 23 °C	2.3	124.2	124.2	2.3	54[b]	4.5	-	
**AR1**	THF, 23 °C	2.3	124.2	124.2	2.3	54[b]	5.1	-	
	THF, 23 °C	2.3	124.2	124.2	2.3	54[b]	4.6±0.8		13±2
[b]5,6-dihydrophenanthridine used instead of HEH_2									
**Figure 2, entry 20: Added [Ir]BAr$^F_4$, subH_2 = 1-benzyl-1,4-dihydronicotinamide**									
**AS1**	THF, 23 °C	2.3	124.2	124.2	2.3	54[c]	1.31	-	
**AT1**	THF, 23 °C	2.3	124.2	124.2	2.3	54[c]	1.12	-	
	THF, 23 °C	2.3	124.2	124.2	2.3	54[c]	1.2±0.1		3.3±0.3
[c]1-benzyl-1,4-dihydronicotinamide used instead of HEH_2									
**Figure 2, entry 21: Added [Ir]BAr$^F_4$, 0.5 atm H_2, 0.5 atm N_2**									
**AU1**	THF, 23 °C $P_{N2} = P_{H2}$ = 0.5 atm	2.3	124.2	124.2	2.3	54	16.1	-	
**AV1**	THF, 23 °C $P_{N2} = P_{H2}$ = 0.5 atm	2.3	124.2	124.2	2.3	54	11.0	-	
	THF, 23 °C $P_{N2} = P_{H2}$ = 0.5 atm	2.3	124.2	124.2	2.3	54	14±4		36±9
**Figure 2, entry 22: Added [Ir(dF(CF_3)ppy)$_2$(dtbbpy)]PF_6 , t = 2 h**									
**AW1**	THF, 23 °C t = 2 h	2.3	124.2	124.2	2.3	54	1.8	-	
**AX1**	THF, 23 °C t = 2 h	2.3	124.2	124.2	2.3	54	2.6	-	
	THF, 23 °C t = 2 h	2.3	124.2	124.2	2.3	54	2.2±0.6		6±1
**Figure 2, entry 23: Added [Ir]PF_6, t = 2 h**									
**AY1**	THF, 23 °C t = 2 h	2.3	124.2	124.2	2.3	54	18.4	-	
**AZ1**	THF, 23 °C t = 2 h	2.3	124.2	124.2	2.3	54	23.5	-	
	THF, 23 °C t = 2 h	2.3	124.2	124.2	2.3	54	21±4		58±10

Figure 2, entry 24: Added [Ir(*p*-F(Me)ppy)$_2$(dtbbpy)]PF_6 , t = 2 h

**BA1**	THF, 23 °C t = 2 h	2.3	124.2	124.2	2.3	54	21.5	-	
**BB1**	THF, 23 °C t = 2 h	2.3	124.2	124.2	2.3	54	23.1	-	
	THF, 23 °C t = 2 h	2.3	124.2	124.2	2.3	54	22±1		62±3

Figure 2, entry 25: Added Ir(ppy)$_3$, t = 2 h

**BC1**	THF, 23 °C t = 2 h	2.3	124.2	124.2	2.3	54	7.8	-	
**BD1**	THF, 23 °C t = 2 h	2.3	124.2	124.2	2.3	54	5.8	-	
	THF, 23 °C t = 2 h	2.3	124.2	124.2	2.3	54	7±1		19±4

Figure 2, entry 26: Added [Ir]BAr^F_4, no Col/[ColH]OTf

**BE1**	THF, 23 °C t = 2 h	2.3	124.2	124.2	2.3	54	7.03	-	
**BF1**	THF, 23 °C t = 2 h	2.3	124.2	124.2	2.3	54	7.83	-	
	THF, 23 °C t = 2 h	2.3	124.2	124.2	2.3	54	7.4±0.4		20.7±1

S1.5.2 Additional catalytic experiments
Table S2 Canvassing H_2 carriers

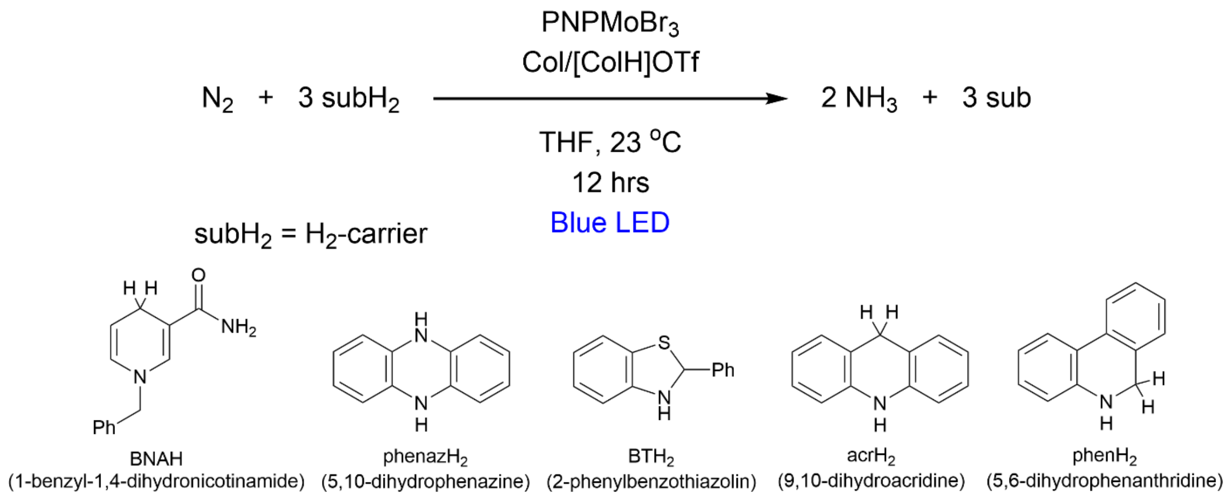

Run	$subH_2$	$[Mo]Br_3$ load (µmol)	acid (µmol)	base (µmol)	Ir (µmol)	$subH_2$ equiv /Mo	NH_3 equiv /Mo	N_2H_4 equiv/Mo	NH_3 yield/ $subH_2$ (%)
**A2**	BNAH	2.3	124.2	124.2	**0**	54	0.55	-	
**B2**	BNAH	2.3	124.2	124.2	**0**	54	0.30	-	
	BNAH	2.3	124.2	124.2	**0**	54	0.4±0.1		1.2±0.3
**C2**	$PhenazH_2$	2.3	124.2	124.2	**2.3**	54	<0.1	-	
**D2**	$PhenazH_2$	2.3	124.2	124.2	**2.3**	54	<0.1	-	
	$PhenazH_2$	2.3	124.2	124.2	**2.3**	54	<0.1		<0.1
**E2**	BTH_2	2.3	124.2	124.2	**2.3**	54	<0.1	-	
**F2**	BTH_2	2.3	124.2	124.2	**2.3**	54	<0.1	-	
	BTH_2	2.3	124.2	124.2	**2.3**	54	<0.1		<0.1
**G2**	$AcrH_2$	2.3	124.2	124.2	**0**	54	0.09	-	
**H2**	$AcrH_2$	2.3	124.2	124.2	**0**	54	0.24	-	
	$AcrH_2$	2.3	124.2	124.2	**0**	54	0.16±0.08		0.5±0.2
**I2**	$PhenH_2$	2.3	124.2	124.2	**0**	54	0.216	-	
**J2**	$PhenH_2$	2.3	124.2	124.2	**0**	54	0.205	-	
	$PhenH_2$	2.3	124.2	124.2	**0**	54	0.211±0.008		0.66±0.02

Table S3. Additional time course experiments

Run	Conditions	Mo loading (µmol)	acid (µmol)	base (µmol)	Ir (µmol)	HEH_2 equiv /Mo	NH_3 equiv /Mo	N_2H_4 equiv/Mo	NH_3 yield/ HEH_2 (%)
**A3**	THF, 23 °C t = 2 h	2.3	124.2	124.2	2.3	54	24.5	-	
**B3**	THF, 23 °C t = 2 h	2.3	124.2	124.2	2.3	54	25.5	-	
	THF, 23 °C t = 2 hours	2.3	124.2	124.2	2.3	54	25±0.5		69.4±1.5
Approximately 100% completion compared to Figure 2, entry 10									
**C3**	THF, rt, 10 min	2.3	124.2	124.2	2.3	54	8.2	-	22.8
Approximately 30% completion compared to Figure 2, entry 10									

Table S4 Catalysis using [ColH]OTf or Col instead of buffered solution

Run	Conditions	Mo loading (µmol)	acid (µmol)	base (µmol)	Ir (µmol)	HEH_2 equiv /Mo	NH_3 equiv /Mo	N_2H_4 equiv/Mo	NH_3 yield/ HEH_2 (%)
**A4**	THF, 23°C	2.3	496.8	**0**	**0**	54	5.8	-	
**B4**	THF, 23°C	2.3	496.8	**0**	**0**	54	5.5	-	
	THF, 23°C	2.3	496.8	**0**	**0**	54	5.65±.15		15.7±0.4
**C4**	THF, 23°C	2.3	**0**	496.8	**0**	54	1.2	-	
**D4**	THF, 23°C	2.3	**0**	496.8	**0**	54	2.0	-	
	THF, 23°C	2.3	**0**	496.8	**0**	54	1.6±0.4		4.7±1.1

Table S5. Solvent screen

$$N_2 + 3\ HEH_2 \xrightarrow[\text{Solvent, Initial temp.}\rightarrow 23\ ^{o}C,\ 12\ hrs,\ \text{Blue LED}]{PNPMoBr_3,\ Ir(ppy)_2(dtbpy)[BAr^F_4],\ Col/[ColH]OTf} 2\ NH_3 + 3\ HE$$

Run	Conditions	Mo loading (μmol)	acid (μmol)	base (μmol)	Ir (μmol)	HEH_2 equiv /Mo	NH_3 equiv /Mo	N_2H_4 equiv/Mo	NH_3 yield/ HEH_2 (%)
**A5**	THF, -78 →23°C	2.3	11.5	11.5	2.3	54	15.47	-	
**B5**	THF, -78 →23°C	2.3	11.5	11.5	2.3	54	16.06	-	
	THF, -78 →23°C	2.3	11.5	11.5	2.3	54	15.7±0.3		44.8±0.8
**C5**	Tol, 23°C	2.3	11.5	11.5	2.3	54	7	-	
**D5**	Tol, 23°C	2.3	11.5	11.5	2.3	54	7.3	-	
	Tol 23°C	2.3	11.5	11.5	2.3	54	7.15±0.15		19.8±0.8
**E5**	Tol, -78 →23°C	2.3	11.5	11.5	2.3	54	13.01	-	
**F5**	Tol, -78 →23°C	2.3	11.5	11.5	2.3	54	14.24	-	
	Tol, -78 →23°C	2.3	11.5	11.5	2.3	54	13.6±0.6		38±2
**G5**	Et_2O, -78 →23°C	2.3	11.5	11.5	2.3	54	4.08	-	
**H5**	Et_2O, -78 → 23°C	2.3	11.5	11.5	2.3	54	3.97	-	
	Et_2O, -78 → 23°C	2.3	11.5	11.5	2.3	54	4.0±0.1		11.2±0.2
**I5**	THF, -78 →23°C	2.3	11.5	11.5	2.3[a]	54	7.4	-	
**J5**	THF, -78 →23°C	2.3	11.5	11.5	2.3[a]	54	11.7	-	
	THF, -78 →23°C	2.3	11.5	11.5	2.3[a]	54	9.6±2		27±7
**K5**	MeCy, 23°C	2.3	124.2	124.2	0	54	<0.1	-	
**L5**	MeCy, 23°C	2.3	124.2	124.2	0	54	<0.1	-	
	MeCy, 23°C						<0.1		<0.3

[a]Ir(ppy)$_3$ used as photosensitizer
Tol = toluene; MeCy = methylcyclohexane

Solubility of reagents:
Collidine: Soluble in THF, Et_2O, Toluene, C_6H_6, MeCy
Collidinium triflate: Soluble in THF, insoluble in Et_2O, Toluene and C_6H_6, MeCy
[Mo]Br$_3$: Soluble in THF, Toluene and C_6H_6. Sparingly soluble in Et_2O, MeCy
HEH_2: Partially soluble in THF, Et_2O, Toluene and C_6H_6. Most soluble in THF, MeCy
[Ir]BAr$^F_4$: Soluble in THF, Et_2O, partially soluble in C_6H_6 and Toluene

Table S6 Attempted catalysis with $[P_3^BFe]BAr^F_4$

Run	Conditions	Fe loading (μmol)	acid (μmol)	base (μmol)	Ir (μmol)	HEH_2 equiv /Fe	NH_3 equiv /Fe	N_2H_4 equiv/Fe	NH_3 yield/ HEH_2 (%)
**A6**	THF, 23°C	2.3	124.2	124.2	2.3	54	<0.1	<0.1	
**B6**	THF, 23°C	2.3	124.2	124.2	2.3	54	<0.1	<0.1	
	THF, 23°C	2.3	124.2	124.2	2.3	54	<0.1	<0.1	<0.1
**C6**	THF, 23°C	2.3	124.2	124.2	0	54	<0.1	<0.1	
**D6**	THF, 23°C	2.3	124.2	124.2	0	54	<0.1	<0.1	
	THF, 23°C	2.3	124.2	124.2	0	54	<0.1	<0.1	<0.1

S1.6 NH_3 detection results from ^{15}N-HEH_2, ^{15}N-Col/^{15}N-[ColH]OTf and $^{15}N_2$ experiments

S1.6.1 $^{15}N_2$ experiments

Catalytic runs done under a $^{15}N_2$ atmosphere were conducted similarly to those under a $^{14}N_2$ atmosphere, with a few differences described below. The loadings were the same as in Figure 2, Entry 1.

Catalysis is performed in the same catalytic tubes as natural abundance experiments, which are charged with precatalyst, HEH_2, [ColH]OTf, and a stirbar in a nitrogen-filled glovebox as described above. After addition of the solids, the tube is then cooled to 77 K in a cold well. The base (Col) is added by micropipette to the frozen tube by opening the Kontes. The Kontes was closed and the tube is kept frozen, then passed out of the glovebox into a liquid N_2 bath. The headspace of the tube is evacuated while still submerged in liquid N_2.

Na/K dried THF is filtered and 1 mL placed into a separate Schlenk tube. The solvent undergoes freeze-pump thaw cycles (3 cycles) and is then vacuum transferred into the catalysis tube. This tube is allowed to warm up briefly and charged with $^{15}N_2$ via vacuum bridge. The tube is refrozen at 77 K and then transferred to a water bath where it thaws and is allowed to stir under Blue LED irradiation for 12 hours.

S1.6.2 ^{15}N-HEH_2, ^{15}N-Col/^{15}N-[ColH]OTf experiments

Catalytic runs were set-up as described in **S1.1** but using either ^{15}N-HEH_2 as H_2-carrier or ^{15}N-Col/[ColH]OTf as buffer using the same conditions as Figure 2, Entry 1.

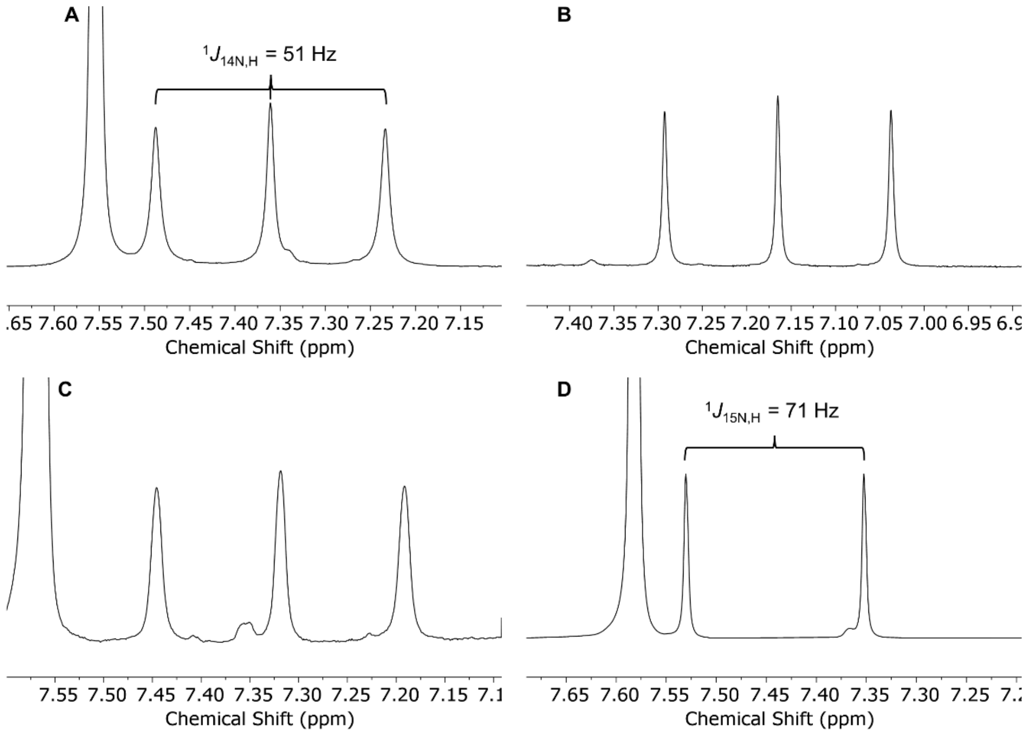

Figure S2. ^{1}H NMR (DMSO-*d*$_6$, 400 MHz) of: A) $^{14}NH_4Cl$ obtained from reaction of natural abundance reactants under $^{14}N_2$ (Ir-free conditions in Figure 2, entry 1); B) $^{14}NH_4Cl$ obtained from reaction of ^{15}N-labeled HEH_2 (otherwise natural abundance reactants) under $^{14}N_2$ (Ir-free conditions in Figure 2, entry 1); C) $^{14}NH_4Cl$ obtained from reaction of ^{15}N-labeled Col/[ColH]OTf (otherwise natural abundance reactants) under $^{14}N_2$ (Ir-free conditions in Figure 2, entry 1); D) $^{15}NH_4Cl$ obtained from reaction under $^{15}N_2$ (otherwise natural abundance reactants, Ir-free conditions in Figure 2, entry 1).

S2.1 Analysis of non-NH_3 catalysis products:

After a complete catalytic run, instead of quenching the reaction (with acid or base) the solvent from the reaction mixture was removed *in vacuo*. Subsequently the resulting film was taken up in minimal solvent (DMSO-d_6, THF-d_8 or 2-MeTHF) and analyzed by NMR or CW-EPR.

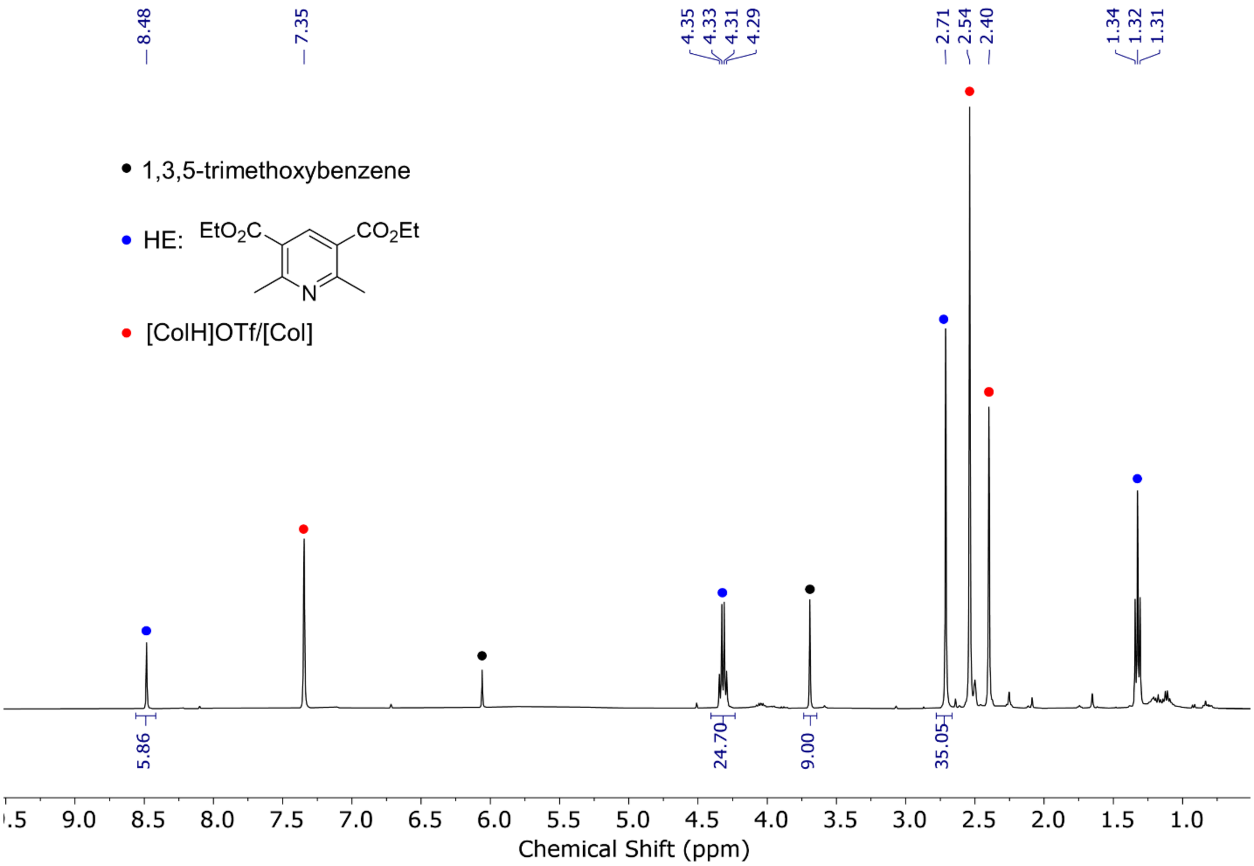

Figure S3. **^{1}H NMR (DMSO-d_6, 400 MHz)** of the nonvolatile products of the Mo-catalyzed reaction of HEH_2, [ColH]OTf, [Col], and N_2 under blue LED irradiation (Figure 2, entry 1).

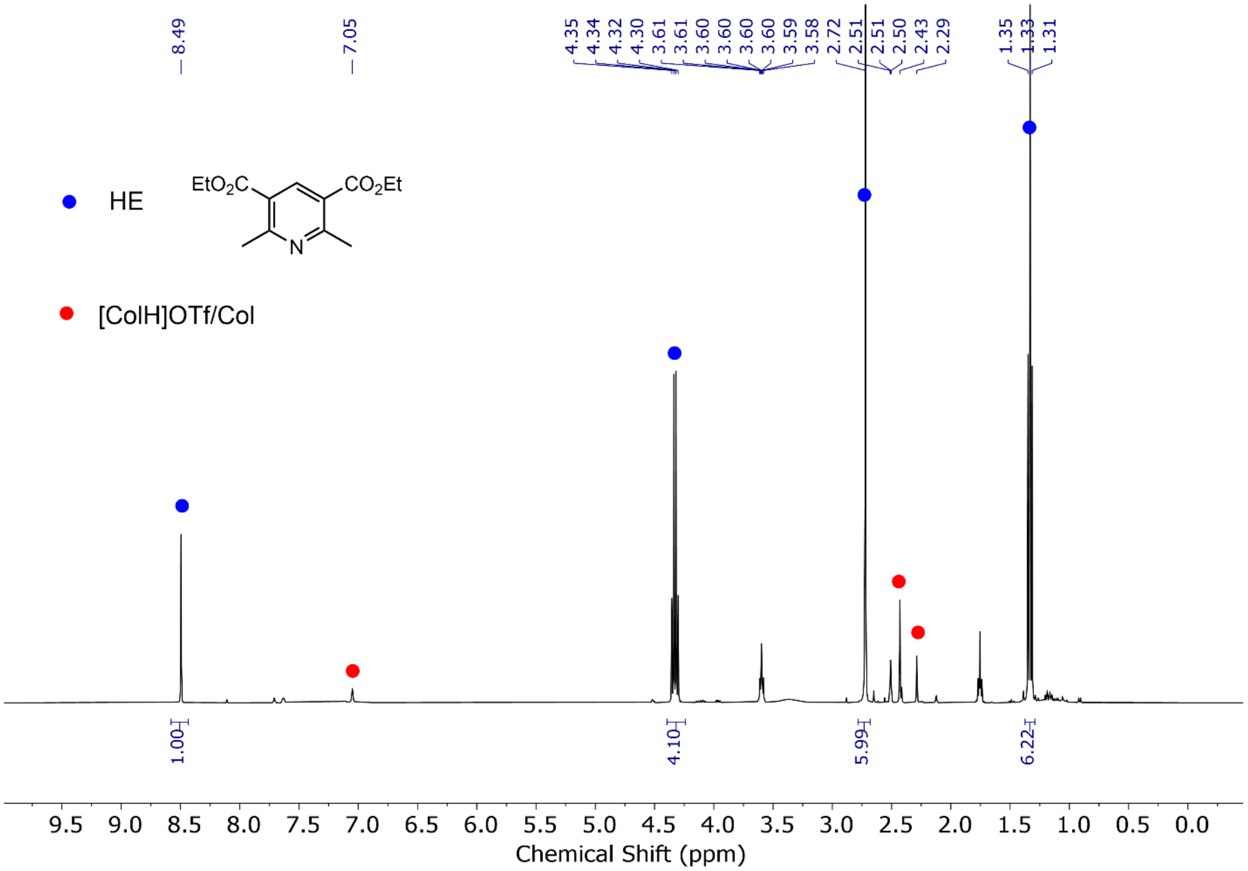

Figure S4. **^{1}H NMR (DMSO-d_6, 400 MHz)** of the nonvolatile products of the Col/[ColH]OTf-, Ir- and Mo-catalyzed reaction of HEH_2 and N_2 under blue LED irradiation (Figure 2, entry 11, catalytic buffer).

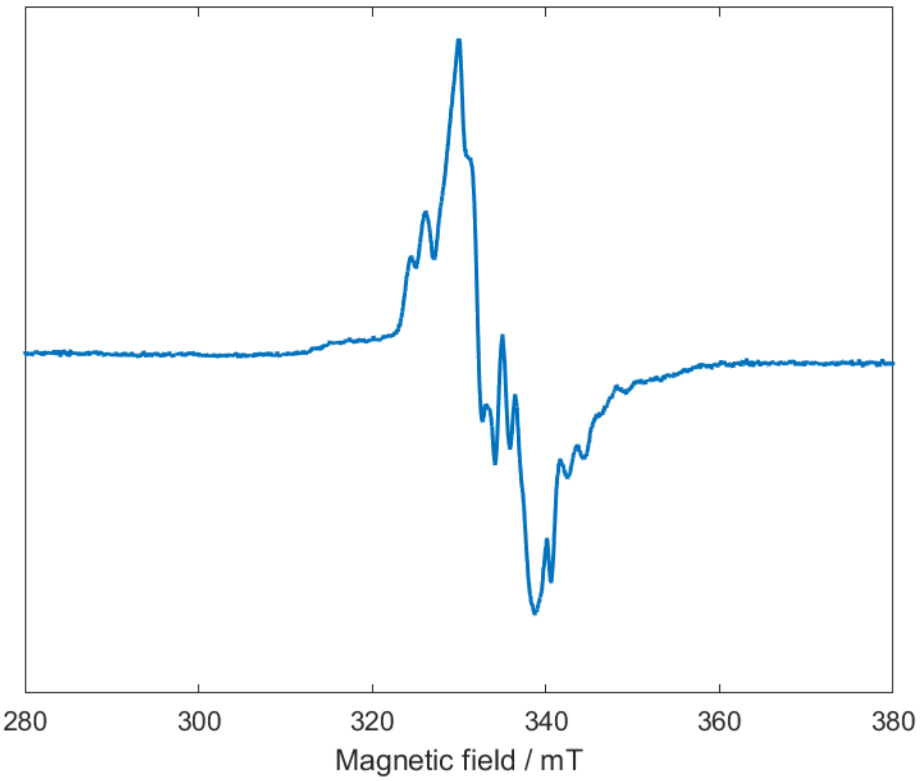

Figure S5. CW-EPR spectrum (2-MeTHF, 77 K) of non-volatile products post-catalysis (conditions in Figure 2, entry 1). Mixture of unknown [Mo] products are observed.

S2.2 ^{1}H NMR time course experiments

Procedure: A J.Young NMR tube was loaded with $[Mo]Br_3$, HEH_2, [ColH]OTf, Col, and N_2 and irradiated under blue LED. Conditions (concentration, temperature) were the same as in Figure 2, entry 1, but using THF-d_8 as solvent and 0.5 mL solvent instead of 1 mL. Slightly slower reaction times are attributed to less efficient illumination of and lack of stirring in the NMR tube compared to Schlenk flasks.

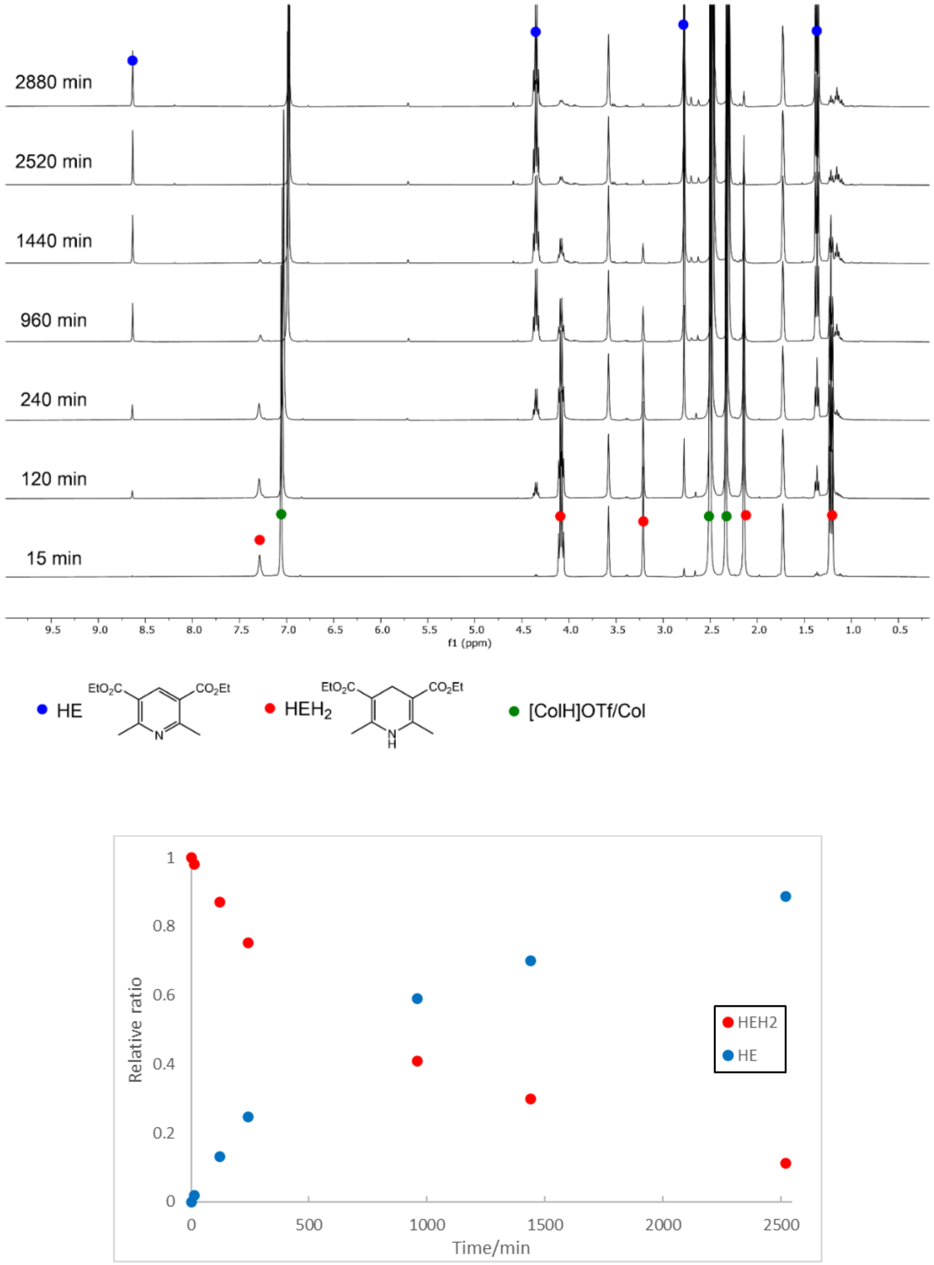

Figure S6. ^{1}**H NMR (THF-d_8, 400 MHz) time course of the Mo-catalyzed reaction of HEH_2, [ColH]OTf, Col, and N_2 under blue LED irradiation in a J.** Young tube (Figure 2, entry 1) (Top). Relative ratio of HE and HEH_2 plotted over time (bottom).

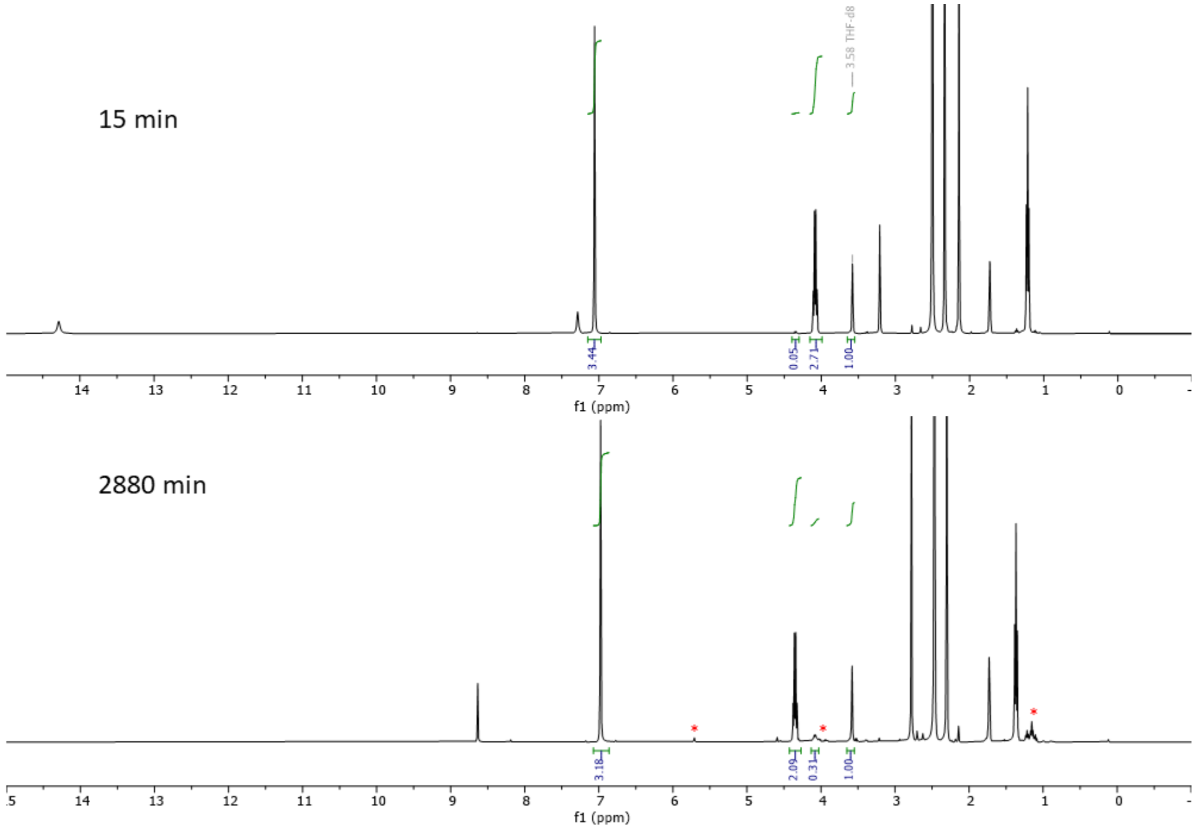

Figure S7. [1]**H NMR (THF-d_8, 400 MHz) of 15 minutes and 48 hour time points of the reaction of the Mo-catalyzed reaction of HEH_2, [ColH]OTf, Col, and N_2 under blue LED irradiation in a J.Young tube (Figure S5).** Integrals of HEH_2 and HE quartet peak at ~ 4.0 ppm are compared to constant THF solvent residual peaks to estimate total recovery of HEH_2 and HE. Approx. 90% is recovered. Similarly, integrals of Col/[ColH]OTf aromatic peak at ~ 7.0 ppm are compared to constant THF solvent residual peaks to estimate total recovery of Col/[ColH]OTf. Approx. 90% is recovered. *indicates minor organic impurity that grows in, see Figure S8.

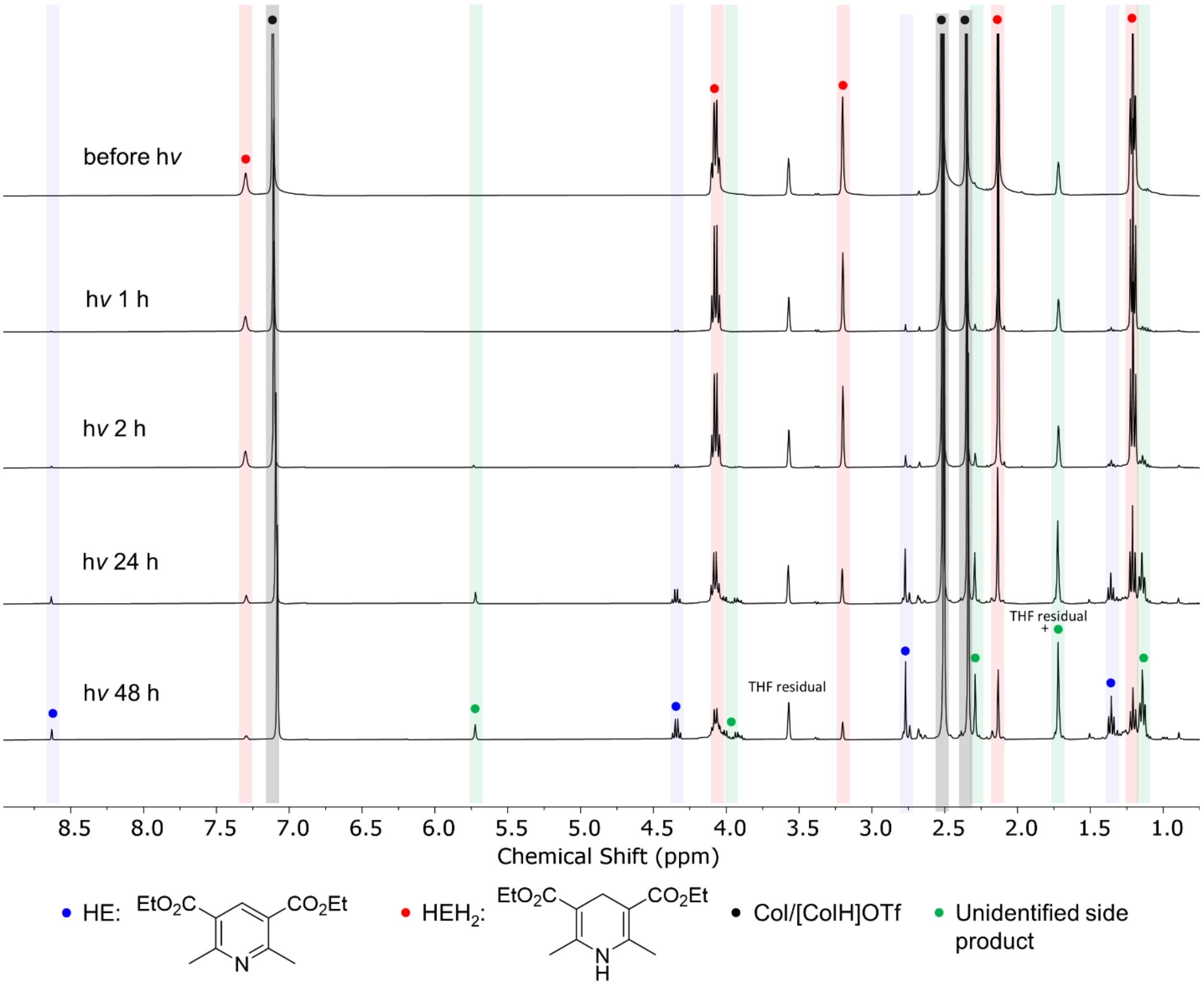

Figure S8. **^{1}H NMR (THF-*d*$_8$, 400 MHz) before irradiation and at indicated timepoints following blue LED irradiation of the reaction of HEH_2 with 1 equiv Col and 1 equiv [ColH]OTf in a J.** Young tube. Integration relative to the THF residual peak at 3.58 ppm indicates that after 48 hours, 79% of HEH_2 and 10% of the total initial buffer loading are consumed, while HE is produced in 16% conversion along with the same major organic side product peaks observed in N_2R with $[Mo]Br_3$ (Figure S6 and S7).

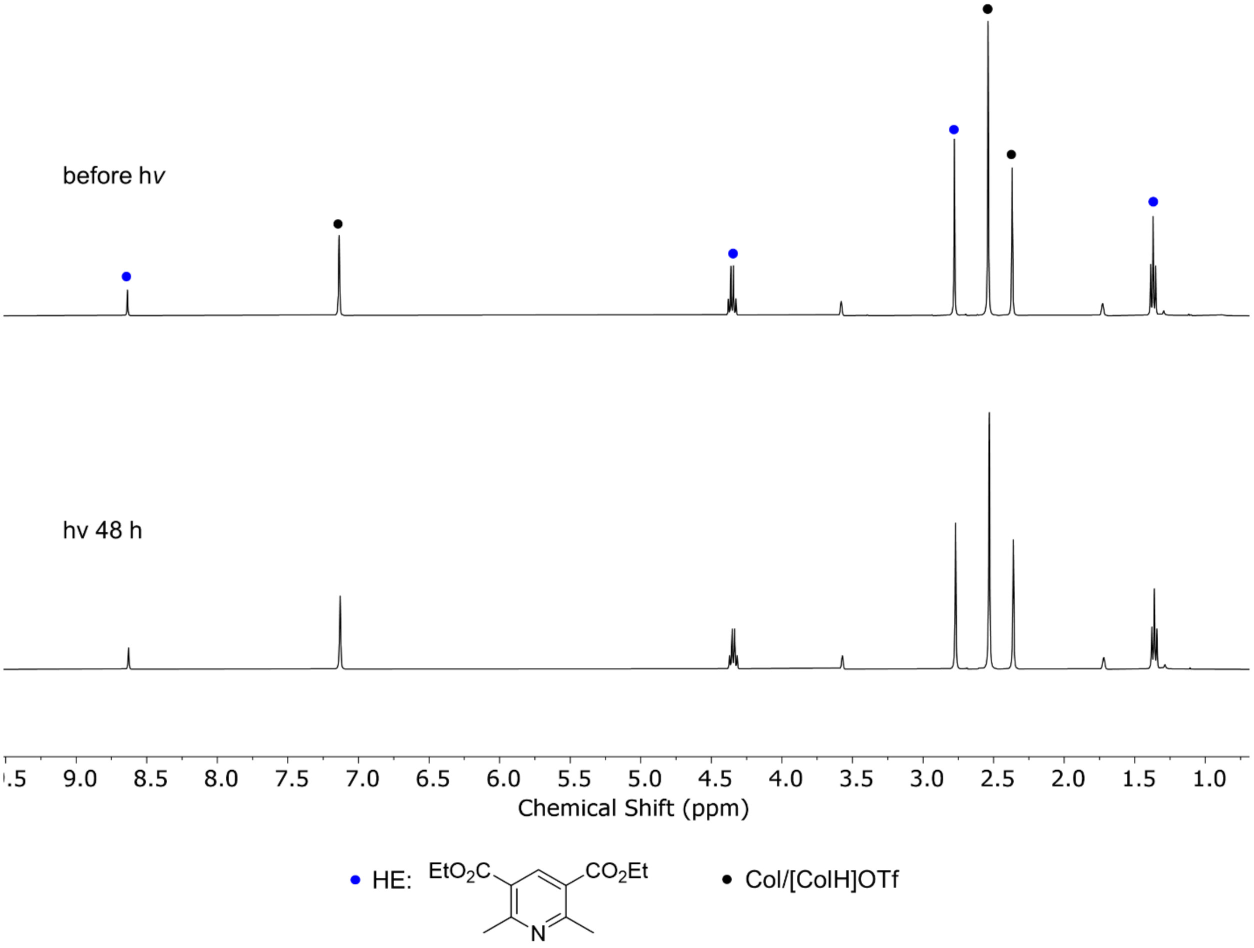

Figure S9. **^{1}H NMR (THF-*d*8, 400 MHz) before irradiation and at indicated timepoints following blue LED irradiation of HE with 1 equiv Col and 1 equiv [ColH]OTf in a J.** Young tube. No reaction is observed.

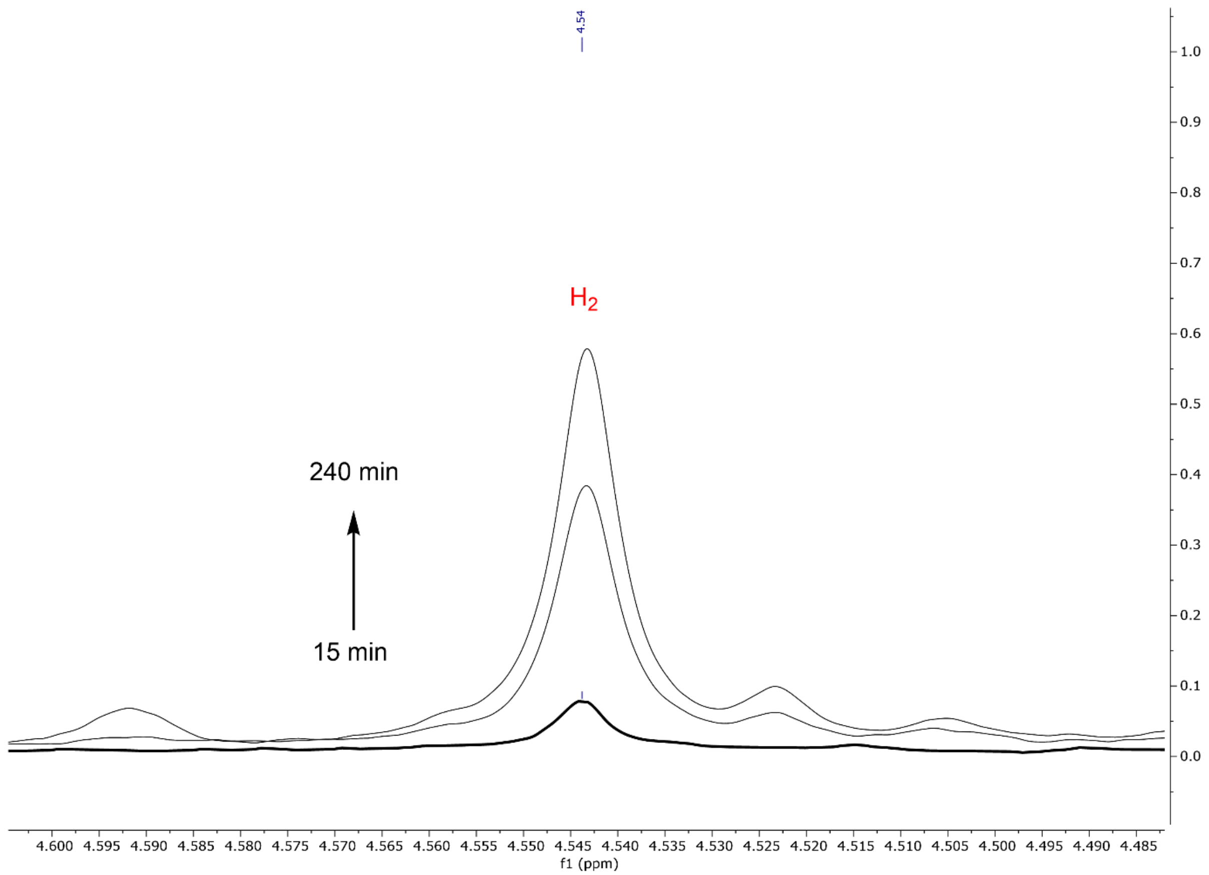

Figure S10. **^{1}H NMR (THF-d_8, 400 MHz) of 15 minutes to 240 min of the Mo-catalyzed reaction of HEH_2, [ColH]OTf, Col, and N_2 under blue LED irradiation in a J.**Young tube (Figure S6). The H_2 peak (4.54 ppm, ref. 45) grows in over time.

S3 Steady-State fluorescence measurements:

S3.1 Fluorimetry studies

Procedure for fluorimetry studies: 1 cm quartz glass cuvettes were loaded with 0.5 mM HEH_2 solutions in dry THF, with varying concentrations of quencher (either Col or [ColH]OTf) in a nitrogen glovebox. Stock solutions were used to assure consistency. Solutions were excited at 390 nm wavelength to avoid interference of the excitation wavelength and steady-state fluorescence spectra. Experiments were conducted at 23 °C.

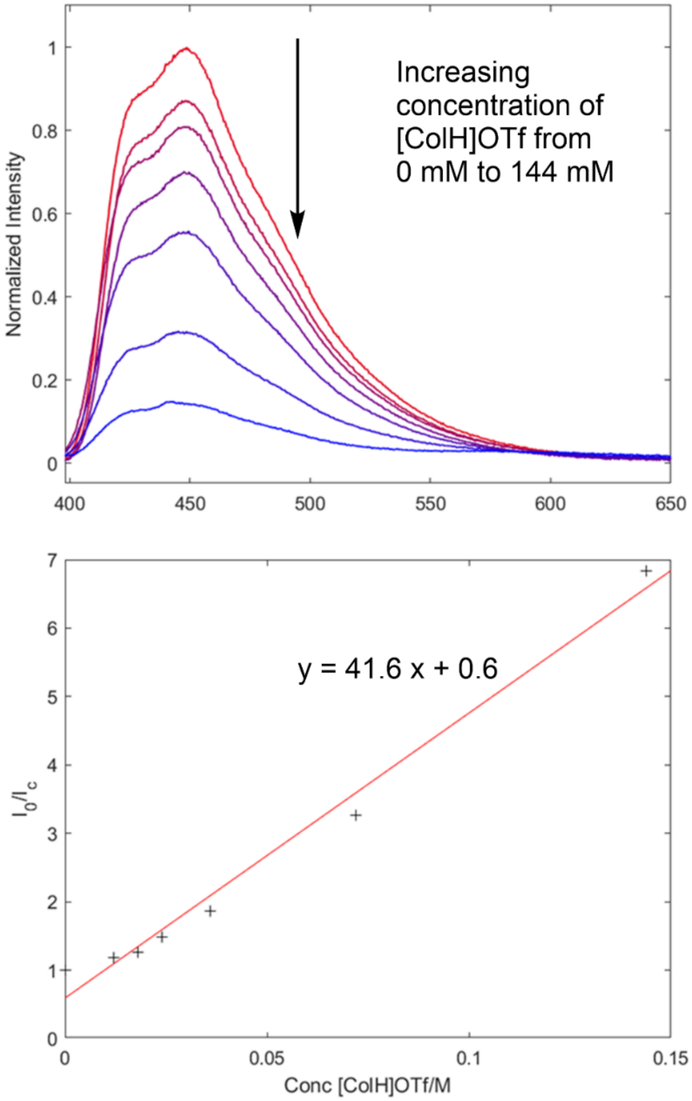

Figure S11. Steady-state fluorescence of HEH_2 (0.5 mM) with varying amounts of [ColH]OTf (18 mM to 144 mM) (Top). Stern-Vollmer quenching plot of I_0/I_C against concentration of [ColH]OTf (bottom). Slope is 42±2.4 M^{-1}; R^2 =0.98.

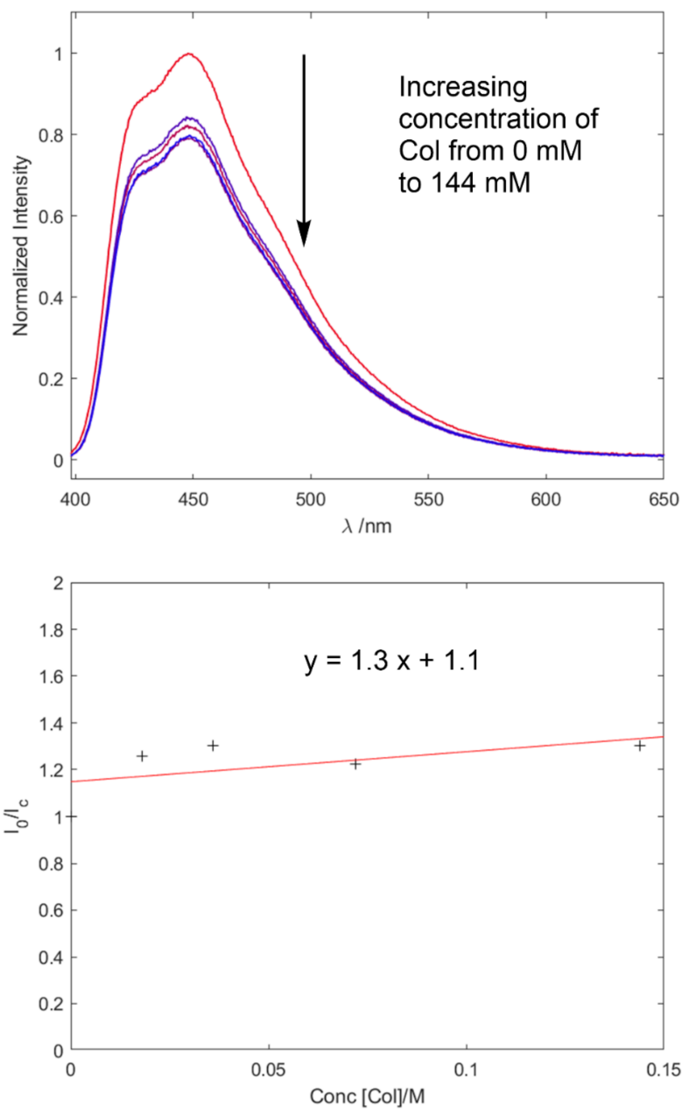

Figure S12. Steady-state fluorescence of HEH_2 (0.5 mM) with varying amounts of Col (18 mM to 144 mM) (Top). Stern-Vollmer quenching plot of I_0/I_C against concentration of Col (bottom). Slope is 1.3±0.9 M^{-1}

S3.2 Calculation of Stern-Volmer quenching constants

Using the previously measured excited state-lifetime measured (τ_0) for HEH_2 we can calculate the Stern-Vollmer quenching lifetime using the equation:

$I_0/I_c = 1 + k_q \cdot \tau_0$ [Q]

k_q = slope/τ_0

While τ_0 has not been measured in THF at 25 °C the measurements in DMSO at 25 °C (0.419 ns) provide a useful estimate.(*20*) Accordingly, the quenching constants are:

$k_{colH} = 1.0 \pm 0.1 \cdot 10^{11}$ M^{-1} s^{-1}

$k_{col} = 3 \pm 2 \cdot 10^{9}$ M^{-1} s^{-1}

While these values have considerable errors, particularly the Col quenching, these nonetheless provide useful order of magnitude estimates. The large rate constant for k_{colH+} suggests the presence of static quenching pathways.

S4 UV-visible measurements

Procedure for UV-vis measurements: 1 cm quartz glass cuvettes were loaded with 0.1 mM HEH_2 solutions in dry THF inside the glovebox. The cuvette was taken out of the glovebox, and spectra were collected. Concentrated (50 mM) solutions of Col or [ColH]OTf were titrated into the cuvettes. The Col or [ColH]OTf solutions had 0.1 mM HEH_2 added to maintain the HEH_2 concentration throughout the experiments. Titrations were done under a sparging N_2 atmosphere to maintain an O_2 free environment. Experiments were conducted at 23 °C.

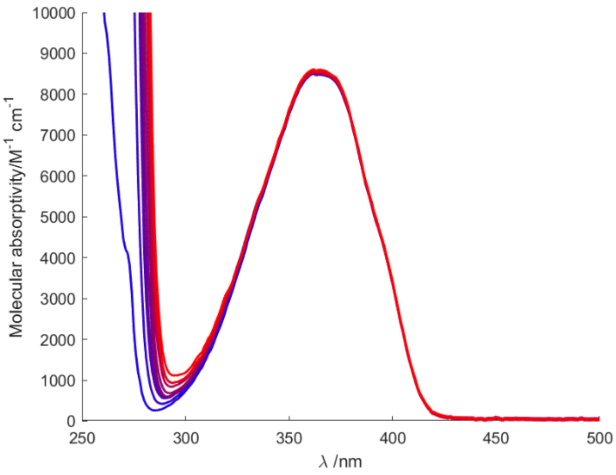

Figure S13. UV-vis of HEH_2 (0.1 mM) with 0 mM (blue) to 14 mM (red) Col concentration.

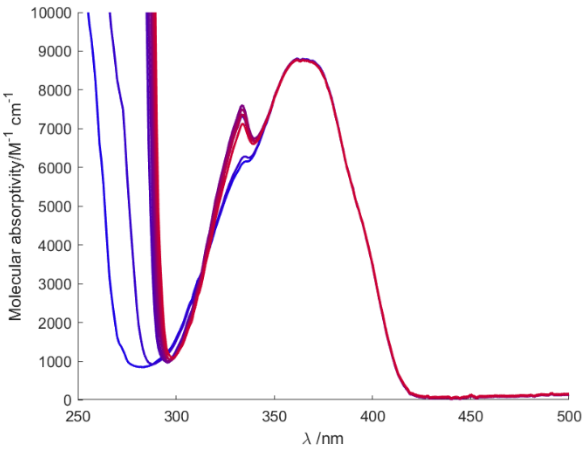

Figure S14. UV-vis of HEH_2 (0.1 mM) with 0 mM (blue) to 11 mM (red) [ColH]OTf concentration.

S5 Reduction of [TBA]NO_3

Proposed balanced equation:

$$[TBA]NO_3 + 4\ HEH_2 + [ColH]OTf \longrightarrow NH_3 + 4\ HE + 3\ H_2O + Col + [TBA]OTf$$

Figure S15. Reaction conditions and balanced equation for the catalytic reduction of [TBA]NO_3 to generate NH_3.

S5.1 Standard [TBA]NO_3 Reduction Generation Reaction Procedure

Catalytic experiments for the reduction of [TBA]NO_3 were conducted in a manner similar to the reduction of N_2 (section **S1.1**). All solvents are stirred with Na/K for ≥2 hours and filtered prior to use. In a nitrogen-filled glovebox, the precatalysts ([Mo]Br_3 and [Ir]BAr^F_4) (2.3 µmol) are weighed in individual vials.* The precatalysts are then transferred quantitatively into a Schlenk tube using THF. The THF is then evaporated to provide a thin film of precatalyst at the bottom of the Schlenk tube. The tube is then charged with a stir bar and the [TBA]NO_3, acid and Hantzsch ester (HEH_2) are added as solids. The tube is cooled to 77 K in a cold well and the base ([Col]) is added as well. The tubes were passed out of the glovebox without warming and thoroughly degassed. 1 mL of degassed (three freeze-pump thaw cycles) THF solvent was vacuum transferred into the catalytic tube. The tube was allowed to warm briefly, and was back-filled with argon. The tube is instead transferred to a water bath where it thaws and is allowed to stir for 12 hours. To ensure reproducibility, all experiments were conducted in 200 mL Schlenk tubes (50 mm OD) using 10 mm eggshaped-stir bars and stirring was conducted at ~600 rpm. The water bath was contained in highly reflective dewars. The Blue LED was placed above the bath as close to the stirring reaction as possible.

S5.2 NH_3 detection

NH_3 was detected by ^1H NMR as detailed in S1.4 NH_3 detection by ^1H NMR.

S5.3 Catalytic reduction of [TBA]NO_3

Table S7. Catalytic yields for photodriven transfer hydrogenation of [TBA]NO_3 to NH_3

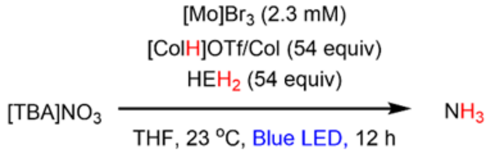

Run	Conditions	[Mo]Br_3 load (µmol)	acid (µmol)	base (µmol)	Ir (µmol)	HEH_2 /Mo	[TBA] NO_3 equiv/ Mo	NH_3 equiv /Mo	NH_3 yield/ HEH_2 (%)
**Figure 2, entry 27: Standard conditions for reduction of [TBA]NO_3**									
**A7**	THF, 23 °C	2.3	124.2	124.2	**0**	54	18	8.5	
**B7**	THF, 23 °C	2.3	124.2	124.2	**0**	54	18	11.0	
	THF, 23 °C	2.3	124.2	124.2	0	54	18	9.8±1.2	73±9
**Figure 2, entry 28: with [Mo]Br_3, with [Ir]BAr^F_4**									
**C7**	THF, 23 °C	2.3	124.2	124.2	**2.3**	54	18	9.9	
**D7**	THF, 23 °C	2.3	124.2	124.2	**2.3**	54	18	10.9	
	THF, 23 °C	2.3	124.2	124.2	**2.3**	54	18	10.4±0.5	77±4
**Figure 2, entry 29: No [Mo]Br_3**									
**E7**	THF, 23 °C	2.3	124.2	124.2	**2.3**	54	18	2	
**F7**	THF, 23 °C	2.3	124.2	124.2	**2.3**	54	18	1.4	
	THF, 23 °C	2.3	124.2	124.2	**2.3**	54	18	1.7±0.3	13±2
**Figure 2, entry 30: No [Mo]Br_3, with [Ir]BAr^F_4**									
**F7**	THF, 23 °C	2.3	124.2	124.2	**2.3**	54	18	3.0	
**G7**	THF, 23 °C	2.3	124.2	124.2	**2.3**	54	18	5.4	
	THF, 23 °C	2.3	124.2	124.2	**2.3**	54	18	4.2±1.2	31±9
**Figure 2, entry 31: no light, with [Mo]Br_3, with [Ir]BAr^F_4**									
**H7**	THF, 23 °C No light	2.3	124.2	124.2	**2.3**	54	18	0.1	
**I7**	THF, 23 °C No light	2.3	124.2	124.2	**2.3**	54	18	0.1	
	THF, 23 °C No light	2.3	124.2	124.2	**2.3**	54	18	0.1±0.05	0.7±0.3

S 5.4 Catalytic reduction of [TBA]$^{15}NO_3$

Catalytic runs were set-up as described in **S5.1** but using [TBA]$^{15}NO_3$.

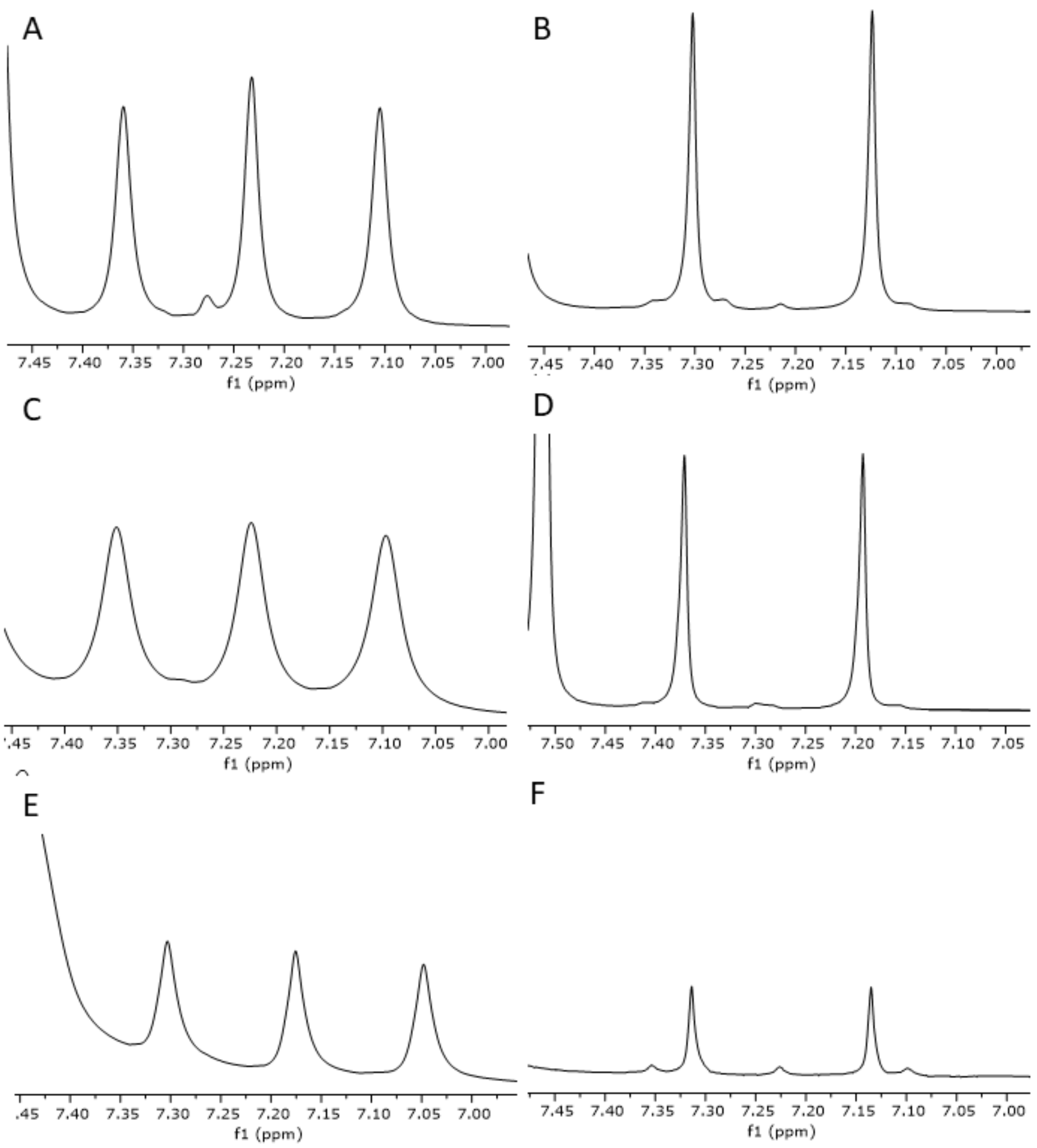

Figure S16. ^{1}H NMR (DMSO-d_6, 400 MHz) of: A) $^{14}NH_4Cl$ obtained from reaction of natural abundance [TBA]NO_3 with HEH_2, buffer, and [Mo]Br_3 under blue light irradiation (Table S7 entry A7); B) $^{15}NH_4Cl$ obtained from reaction of [TBA]$^{15}NO_3$ with HEH_2, buffer, and [Mo]Br_3 under blue light irradiation; C) $^{14}NH_4Cl$ obtained from reaction of natural abundance [TBA]NO_3 with HEH_2, buffer, [Mo]Br_3 and [Ir]$BAr^F{}_4$ under blue light irradiation (Table S7, entry C7); D) $^{15}NH_4Cl$ obtained from reaction of [TBA]$^{15}NO_3$ with HEH_2, buffer, [Mo]Br_3 and [Ir]$BAr^F{}_4$ under blue light irradiation; E) $^{14}NH_4Cl$ obtained from reaction of natural abundance [TBA]NO_3 with HEH_2, buffer, and [Ir]$BAr^F{}_4$ under blue light irradiation (Table S7, entry F7); F) $^{15}NH_4Cl$ obtained from reaction of [TBA]$^{15}NO_3$ with HEH_2, buffer, and [Ir]$BAr^F{}_4$ under blue light irradiation.

S5.5 Comment on nitrate reduction in the absence of light or [Mo]Br$_3$.

It is worth commenting on the fact that [TBA]NO$_3$ reduction can occur both in the absence of light and [Mo]Br$_3$, albeit with diminished yields. This differs from N$_2$R where both are required and no NH$_3$ can be detected. Nitrate differs as a substrate from N$_2$, in that it is more activated, and forms relatively stable intermediates during reduction (NO$_2^-$, NO), and the thermodynamics of reduction are more favorable. This is illustrated in Figure S17 showing the thermodynamics between different intermediates in the reduction of N$_2$ and NO$_3^-$ (in aqueous solution, vs NHE). Therefore, a molecular catalyst might not be required to activate the substrate prior to reduction/protonation and stabilize intermediates that form during reduction. The role of [Mo]Br$_3$ might therefore be primarily as a Lewis acid or a solubilizing agent. Ultimately, these results suggest that higher yields/efficiencies and possibly even nitrate reduction without illumination may all be possible with a more careful choice of catalyst and warrant further exploration.

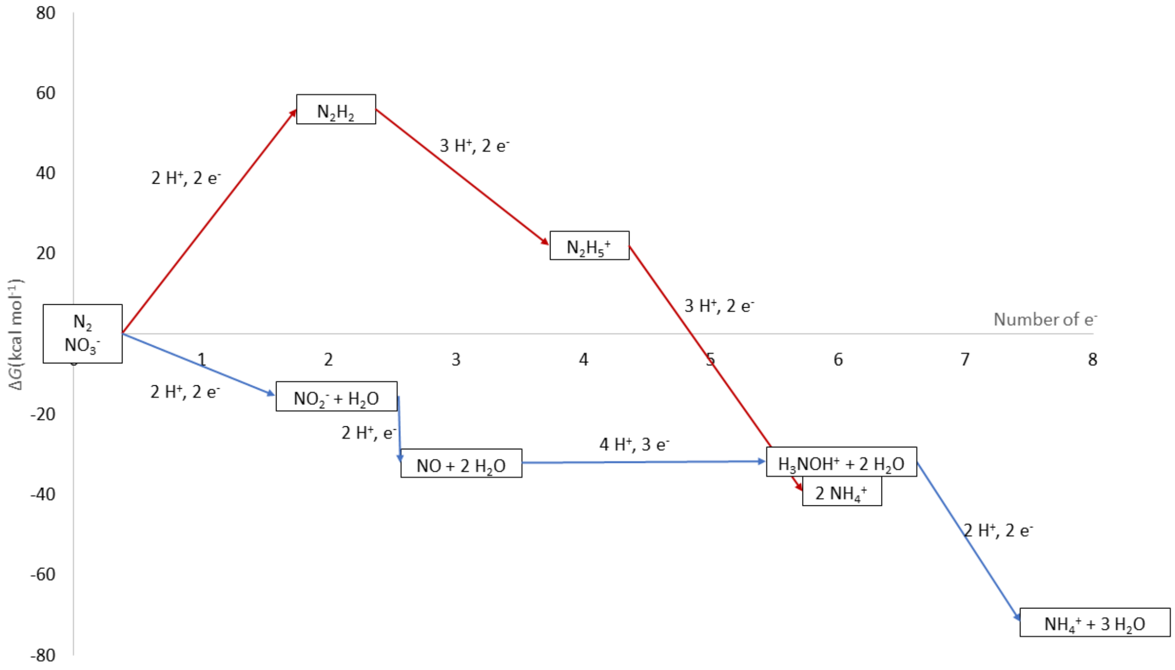

Figure S17: Comparison of Δ*G* (in kcal mol^{-1}) in aqueous solution of pH 0 referenced to NHE.(*49, 50*) While the 6 e$^-$ reduction and 8 e$^-$ reduction of N$_2$ and NO$_3^-$ respectively are both downhill, only the intermediates of NO$_3^-$ are also thermodynamically favored to form.

S6 Reduction of acetylene:

Proposed balanced equations:

$$C_2H_2 + HEH_2 \longrightarrow C_2H_4 + HE$$

$$C_2H_4 + HEH_2 \longrightarrow C_2H_6 + HE$$

Figure S18. Balanced equations for the catalytic reduction of acetylene to generate ethylene and ethane.

S6.1 Standard acetylene reduction reaction procedure

Catalytic experiments for the reduction of acetylene were conducted in a manner similar to the reduction of N_2 (section **S1.1**). All solvents are stirred with Na/K for ≥2 hours and filtered prior to use. In a nitrogen-filled glovebox, the precatalysts ([Mo]Br_3 and [Ir]BAr^F_4) (2.5 µmol) are weighed in individual vials. The precatalysts are then transferred quantitatively into a Schlenk tube using THF. The THF is then evaporated to provide a thin film of precatalyst at the bottom of the Schlenk tube. The tube is then charged with a stirbar and [ColH]OTf and Hantzsch ester (HEH_2) are added to the vial as solids. The tube is wrapped in aluminum foil and Col and THF-d_8 (0.7 mL) are added. The tube is sealed, passed out of the glovebox, and degassed (three freeze-pump thaw cycles). The desired volume of acetylene gas is added using a calibrated bulb while the tube is cooled in liquid nitrogen. The headspace of the tube is then backfilled to 1 atm with argon while cooled in a dry ice/acetone bath. The tube is transferred to a water bath and is irradiated with Blue LED for the time specified. The water bath was contained in highly reflective dewars. The Blue LED was placed above the bath as close to the reaction as possible.

After 12 hours of irradiation, the volatiles of the reaction mixture are vacuum transferred into a J. Young NMR tube of known volume containing a known amount of 1,3,5-trimethoxybenzene. In the ^{1}H NMR spectrum of the resulting sample, the peaks corresponding to ethylene (5.36 ppm) and ethane (0.85 ppm) are clearly distinguishable when present.(*45*) Integration to the internal standard provides the yield of dissolved gases. Henry's constant for each gas in THF(*48*) was used to estimate their partial pressures in the headspace.

S6.2 Ethylene and ethane detection results

Table S8: Catalytic yields for photodriven transfer hydrogenation of acetylene to ethylene and ethane.

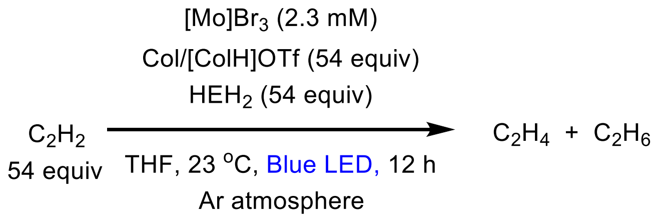

Run	Conditions	[Mo] load (µmol)	acid (µmol)	base (µmol)	Ir (µmol)	HEH_2 equiv /Mo	C_2H_4 equiv /Mo	C_2H_6 equiv/ Mo	Total yield/ HEH_2 (%)
**Figure 2, entry 32: standard conditions**									
**A8**	THF, 23 °C	2.5	135	135	0	54	8.2	1.3	
**B8**	THF, 23 °C	2.5	135	135	0	54	11.4	1.7	
	THF, 23 °C	2.5	135	135	0	54	10±2	1.5±0.3	24±5
**Figure 2, entry 33: with [Mo]Br₃, with [Ir]BAr$^F_4$**									
**C8**	THF, 23 °C	2.5	135	135	2.5	54	6.4	1.3	
**D8**	THF, 23 °C	2.5	135	135	2.5	54	4.9	0.9	
	THF, 23 °C	2.5	135	135	2.5	54	6±1	1.1±0.3	15±3
**Figure 2, entry 34: no [Mo]Br₃, no [Ir]BAr$^F_4$**									
**E8**	THF, 23 °C	2.5	135	135	0	54	0.048	<0.03	
**F8**	THF, 23 °C	2.5	135	135	0	54	0.059	<0.03	
	THF, 23 °C	2.5	135	135	0	54	0.054± 0.008	<0.03	<0.3
**Figure 2, entry 35: no [Mo]Br₃, with [Ir]BAr$^F_4$**									
**G8**	THF, 23 °C	0	135	135	2.5	54	0.8	0.02	
**H8**	THF, 23 °C	0	135	135	2.5	54	3.0	0.14	
	THF, 23 °C	0	135	135	2.5	54	2±2	0.08± 0.08	4±3
**Figure 2, entry 36: with [Mo]Br₃, with [Ir]BAr$^F_4$, no light**									
**I8**	THF, 23 °C no light	2.5	135	135	2.5	54	<0.01	<0.01	
**J8**	THF, 23 °C no light	2.5	135	135	2.5	54	<0.01	<0.01	
	THF, 23 °C no light	2.5	135	135	2.5	54	<0.01	<0.01	<0.04

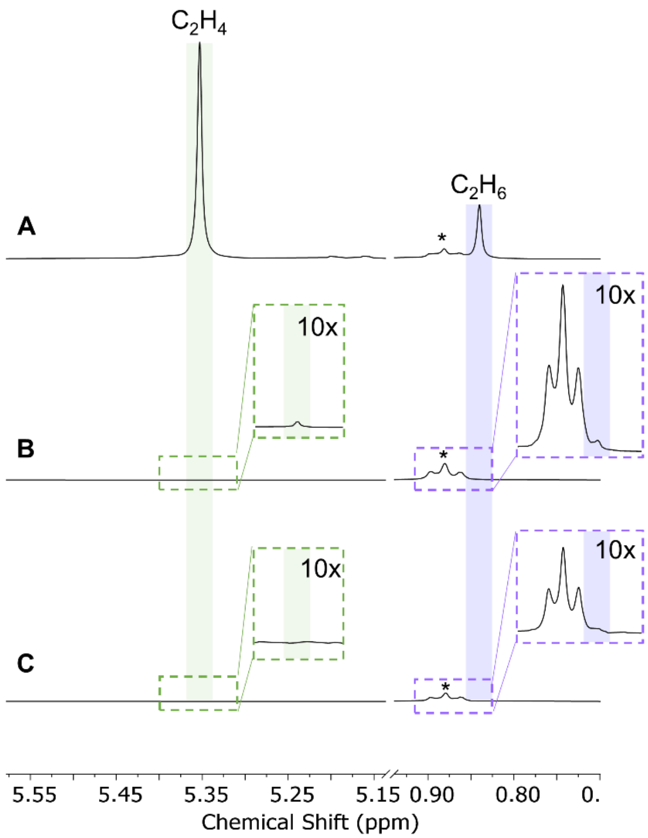

Figure S19. ^{1}**H NMR (THF-*d*$_8$, 400 MHz) of the volatiles obtained from the acetylene reduction reaction in Table S8:** A) Standard conditions (Table S8, entry A8); B) No [Mo]Br_3 (Table S8, entry E8); C) No irradiation (Table S8, entry I8). *Trace pentane.

S7. Additional mechanistic schemes

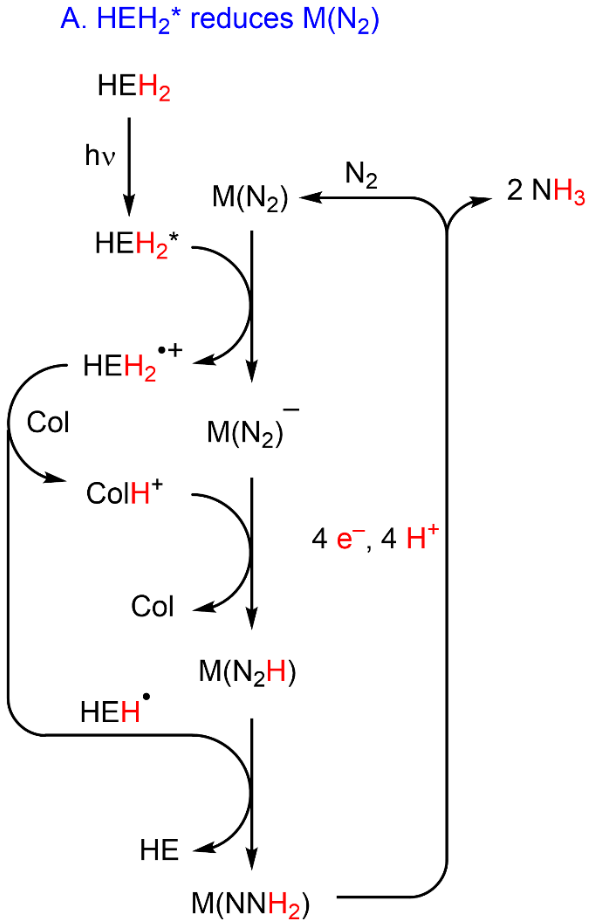

Figure S20. Mechanistic scenario in absence of photoredox catalyst in which $[HEH_2]^*$ is quenched by a $M(N_2)$ intermediate.

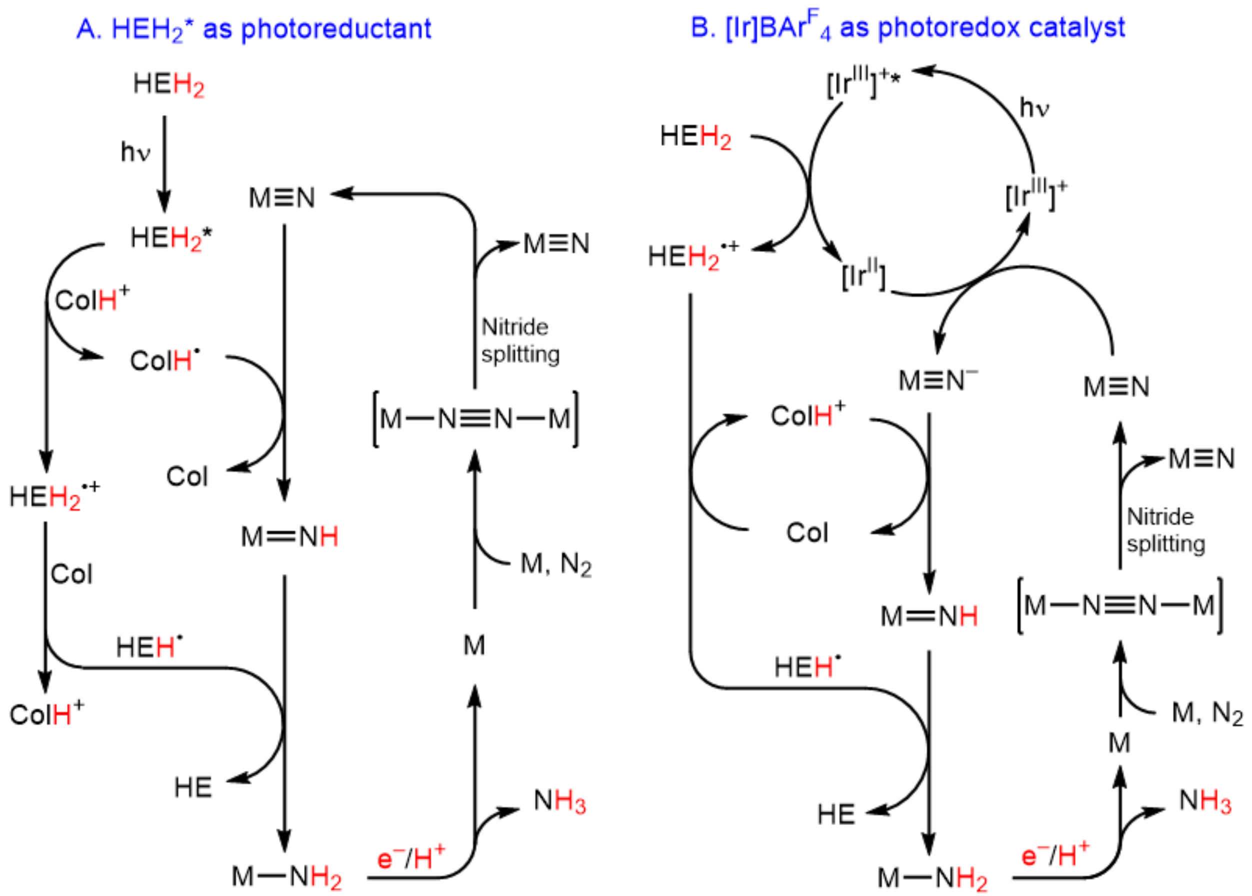

Figure S21. Possible scenarios for photodriven transfer hydrogenation from HEH_2 to N_2 mediated by a metal catalyst and buffer system (Col/$[ColH]^+$). These schemes depict a mechanism in which N_2 cleavage occurs and the subsequent M≡N is hydrogenated. (A) Scenario in absence of photoredox catalyst, in which $[HEH_2]^*$ is oxidatively quenched by $[ColH]^+$ to generate $[ColH]^•$. (B) Scenario with photoredox catalyst, in which $[Ir^{III}]^{+*}$ is reductively quenched by HEH_2.

S8 Derivation of thermodynamic values:

S8.1 Summary of thermochemistry of Hantzsch ester (HEH_2) and derivatives

Table S9 lists $BDFE_{X–H}$, pK_a, and E_{ox} values for various protonation and oxidation states of HEH_2. As has been established by Mayer and coworkers,(*36*) bond dissociation enthalpies (BDEs) can be converted to BDFEs based on the assumption that the entropies of R–H and $R^•$ are similar. Subtraction of $TS°(H^•)_{solv}$ (6.37 kcal mol^{-1} in MeCN) from the BDE values reported in ref. 18 yields the estimated BDFE values in Table S9. With these values and reported potentials of oxidation, relevant pK_a values were then estimated using the thermodynamic cycles laid out below.

	**BDE**^{a}	**BDFE**^{a}	$E_{ox}{}^b$	**pK_a**
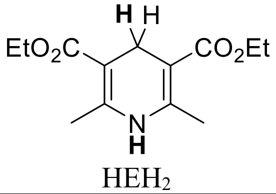 HEH_2	68.7 (C–H), 86.6 (N–H)^c	62.3 (C–H), 80.2 (N–H)	0.48^c	31.8 (N–H)
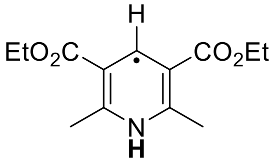 $HEH^•$	46.9 (N–H)^c	40.5 (N–H)		
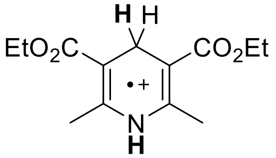 $HEH^{•+}$				–1.0 (C–H)
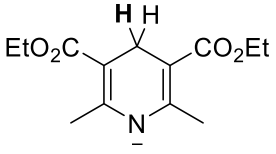 $HEH^–$			–0.695^c	
$[HEH_2]^*$		–8.5 (C–H)^d	–2.6^d	–20(N–H)^d

Table S9. Reported and estimated thermochemical values for various protonation and oxidation states of HEH_2 relevant to this study. ^{a} kcal mol^{-1} in MeCN at 298 K. ^{b} V vs. $Fc^{+/0}$ in MeCN at 298 K. ^{c} Ref. 18. ^{d} Estimated using the E_{00} reported in ref. 19.

Estimation of the N–H pK_a of HEH_2:

$HEH^• + e^- \rightleftharpoons HEH^-$	$-23.06(E_{ox}(HEH^-)) = 16$ kcal mol^{-1}
$H^• \rightleftharpoons H^+ + e^-$	$-C_G = -52.6$ kcal mol^{-1}
$HEH_2 \rightleftharpoons HEH^• + H^•$	$BDFE_{N–H}(HEH_2) = 80.2$ kcal mol^{-1}
$HEH_2 \rightleftharpoons HEH^- + H^+$	1.37(pK_a)
	p$K_{a,N–H}(HEH_2)$ = **31.8**

Estimation of the C–H pK_a of $HEH_2^{\bullet+}$:

$HEH_2^{\bullet} + e^- \rightleftharpoons HEH_2$	$-23.06(E_{ox}(HEH_2)) = -11.1$ kcal mol^{-1}
$H^{\bullet} \rightleftharpoons H^+ + e^-$	$-C_G = -52.6$ kcal mol^{-1}
$\underline{HEH_2 \rightleftharpoons HEH^{\bullet} + H^{\bullet}}$	$\underline{BDFE_{C-H}(HEH_2) = 62.3 \text{ kcal mol}^{-1}}$
$HEH_2^{\bullet+} \rightleftharpoons HEH^{\bullet} + H^+$	$1.37(pK_a)$
	$pK_{a,C-H}(HEH^{\bullet+}) = \mathbf{-1.0}$

Estimation of the excited-state N–H pK_a of $[HEH_2]^*$:

$HEH_2^* \rightleftharpoons HEH_2$	$-23.06(E_{00}) = -70.8$ kcal mol^{-1}
$\underline{HEH_2 \rightleftharpoons HEH^- + H^+}$	$\underline{1.37(pK_{a,N-H}(HEH_2)) = 43.6 \text{ kcal mol}^{-1}}$
$HEH_2^* \rightleftharpoons HEH^- + H^+$	$1.37(pK_a)$
	$pK_{a,N-H}(HEH_2^*) = \mathbf{-20}$

Estimation of the excited-state $BDFE_{C-H}$ of $[HEH_2]^*$:

$HEH_2^* \rightleftharpoons HEH_2$	$-23.06(E_{00}) = -70.8$ kcal mol^{-1}
$\underline{HEH_2 \rightleftharpoons HEH^- + H^+}$	$\underline{BDFE_{C-H}(HEH_2) = 62.3 \text{ kcal mol}^{-1}}$
$HEH_2^* \rightleftharpoons HEH^- + H^+$	$BDFE_{C-H}(HEH_2^*) = \mathbf{-8.5\ kcal\ mol^{-1}}$

S8.2 Derivation of effective BDFE values ($BDFE_{eff}$) relevant to this work

S8.2.1 Derivation of $BDFE_{eff}$ for $subH_2$ donors

Estimation of $BDFE_{eff}$ for HEH_2:

½ $HEH_2 \rightleftharpoons$ ½ $HEH^{\bullet}$ + ½ $H^{\bullet}$	½ $BDFE_{C–H}(HEH_2)$ = 31.2 kcal mol^{-1}
½ $HEH^{\bullet} \rightleftharpoons$ ½ HE + ½ $H^{\bullet}$	½ $BDFE_{N–H}(HEH^{\bullet})$ = 20.3 kcal mol^{-1}
½ $HEH_2 \rightleftharpoons$ ½ HE + $H^{\bullet}$	$BDFE_{eff}(HEH_2)$ = **51.4 kcal mol^{-1}**

Estimation of $BDFE_{eff}$ for $acrH_2$:

$acrH_2 \rightleftharpoons acrH_2^{\bullet +} + e^-$	$23.06(E_{ox}(acrH_2))$ = 11.3 kcal mol^{-1} (Ref 50)
$acrH_2^{\bullet +} \rightleftharpoons acrH^+ + H^{\bullet}$	$BDE(acrH_2^{\bullet +}) - TS°(H^{\bullet})_{solv}$ = 43.5 – 6.37 kcal mol^{-1} = 37.1 kcal mol^{-1} (Ref 51)
$e^- + H^{\bullet} \rightleftharpoons H^-$	$\Delta G°$ = 26.0 kcal mol^{-1} (Ref 52)
$acrH_2 \rightleftharpoons acrH^+ + H^-$	$\Delta G(acrH_2)_{H^-}$ = **74.4 kcal mol^{-1}**

½ $acrH_2 \rightleftharpoons$ ½ $acrH^+$ + ½ H^-	½ $\Delta G(acrH_2)_{H^-}$ = 37.2 kcal mol^{-1}
½ $acrH^+ \rightleftharpoons$ ½ acr + ½ H^+	½ $1.37(pK_a)$ = 8.7 kcal mol^{-1} (Ref 22)
½ H^- + ½ $H^+ \rightleftharpoons$ ½ H_2	½ $\Delta G°$ = −38 kcal mol^{-1} (Ref 52)
½ $H_2 \rightleftharpoons H^{\bullet}$	½ $BDFE_{N–H}(H_2)$ = 52 kcal mol^{-1} (See S10.3)
½ BNAH + ½ $[ColH]^+ \rightleftharpoons$ ½ BNA + ½ Col + $H^{\bullet}$	$BDFE_{eff}(acrH_2)$ = **59.9 kcal mol^{-1}**

Estimation of $BDFE_{eff}$ for $phenH_2$:

½ $phenH_2 \rightleftharpoons$ ½ $phenH^{\bullet}$ + ½ $H^{\bullet}$	½ $BDFE_{C–H}(phenH_2)$ = 33.1 kcal mol^{-1} (Ref 18)
½ $phenH^{\bullet} \rightleftharpoons$ ½ phen + ½ $H^{\bullet}$	½ $BDFE_{N–H}(phenH^{\bullet})$ = 19.1 kcal mol^{-1} (Ref 18)
½ $phenH_2 \rightleftharpoons$ ½ phen + $H^{\bullet}$	$BDFE_{eff}(phenH_2)$ = **52.2 kcal mol^{-1}**

Estimation of $BDFE_{eff}$ for BNAH:

BNAH is a 1 H^+ /2 e^- donor. As such to balance the equation for the 6 H^+/6 e^- reduction of N_2 we posit that [ColH]OTf must also be consumed giving a balanced reaction:

N_2 + 3 BNAH + 3 $[ColH]^+ \rightarrow NH_3$ + 3 $[BNA]^+$ + 3 Col

To estimate $BDFE_{eff}$ we instead combine the hydricity of BNAH and the acidity of [ColH]OTf

½ BNAH $\rightleftharpoons$ ½ BNA + ½ H^-	½ $\Delta G(BNAH)_{H^-}$ = 29.5 kcal mol^{-1} (Ref 53)
½ $[ColH]^+ \rightleftharpoons$ ½ Col + ½ H^+	½ $1.37(pK_a)$ = 10.3 kcal mol^{-1} (Ref 22)
½ H^- + ½ $H^+ \rightleftharpoons$ ½ H_2	½ $\Delta G°$ = −38 kcal mol^{-1} (Ref 52)
½ $H_2 \rightleftharpoons H^{\bullet}$	½ $BDFE_{N–H}(H_2)$ = 52 kcal mol^{-1} (See S10.3)
½ BNAH + ½ $[ColH]^+ \rightleftharpoons$ ½ BNA + ½ Col + $H^{\bullet}$	$BDFE_{eff}(BNAH/ColH^+)$ = **53.8 kcal mol^{-1}**

S8.2.2 Derivation of $BDFE_{eff}$ for reductant (photosensitizer or *HEH_2) and acid

Estimation of $BDFE_{eff}$ for $[HEH_2]^*$ as reductant and $[ColH]^+$ as acid:

$HEH_2^* \rightleftharpoons HEH_2^{\bullet+} + e^-$
$[ColH]^+ \rightleftharpoons Col + H^+$ — $1.37(pK_a) = 20.6$ kcal mol^{-1} (Ref 22)
$\underline{H^+ + e^- \rightleftharpoons H^\bullet}$ — $\underline{C_G = 52.6 \text{ kcal mol}^{-1}}$
$HEH_2^* + [ColH]^+ \rightleftharpoons HEH_2^{\bullet+} + Col + H^\bullet$ — $BDFE_{eff}$ = **10.9 kcal mol^{-1}**

Estimation of $BDFE_{eff}$ for $Ir^{II}(ppy)_2(dtbbpy)$ as reductant and $[ColH]^+$ as acid:

$[Ir^{II}] \rightleftharpoons [Ir^{III}]^+ + e^-$ — $23.06(E_{ox}([Ir^{II}]) = -43.8$ kcal mol^{-1} (Ref 28)
$[ColH]^+ \rightleftharpoons Col + H^+$ — $1.37(pK_a) = 20.6$ kcal mol^{-1} (Ref 22)
$\underline{H^+ + e^- \rightleftharpoons H^\bullet}$ — $\underline{C_G = 52.6 \text{ kcal mol}^{-1}}$
$HEH_2^* + [ColH]^+ \rightleftharpoons HEH_2^{\bullet+} + Col + H^\bullet$ — $BDFE_{eff}$ = **29.3 kcal mol^{-1}**

Estimation of $BDFE_{eff}$ for Ir^{II}(*p*-F(Me)ppy)$_2$(dtbbpy) as reductant and $[ColH]^+$ as acid:

$[Ir^{II}] \rightleftharpoons [Ir^{III}]^+ + e^-$ — $23.06(E_{ox}([Ir^{II}]) = -43.4$ kcal mol^{-1} (Ref 30)
$[ColH]^+ \rightleftharpoons Col + H^+$ — $1.37(pK_a) = 20.6$ kcal mol^{-1} (Ref 22)
$\underline{H^+ + e^- \rightleftharpoons H^\bullet}$ — $\underline{C_G = 52.6 \text{ kcal mol}^{-1}}$
$HEH_2^* + [ColH]^+ \rightleftharpoons HEH_2^{\bullet+} + Col + H^\bullet$ — $BDFE_{eff}$ = **29.8 kcal mol^{-1}**

Estimation of $BDFE_{eff}$ for $Ir^{II}(dF(CF_3)ppy)_2(dtbbpy)$ as reductant and $[ColH]^+$ as acid:

$[Ir^{II}] \rightleftharpoons [Ir^{III}]^+ + e^-$ — $23.06(E_{ox}([Ir^{II}]) = -40.4$ kcal mol^{-1} (Ref 29)
$[ColH]^+ \rightleftharpoons Col + H^+$ — $1.37(pK_a) = 20.6$ kcal mol^{-1} (Ref 22)
$\underline{H^+ + e^- \rightleftharpoons H^\bullet}$ — $\underline{C_G = 52.6 \text{ kcal mol}^{-1}}$
$HEH_2^* + [ColH]^+ \rightleftharpoons HEH_2^{\bullet+} + Col + H^\bullet$ — $BDFE_{eff}$ = **32.8 kcal mol^{-1}**

Estimation of $BDFE_{eff}$ for $[Ir^{II}(ppy)_3]^-$ as reductant and $[ColH]^+$ as acid:

$[Ir^{II}] \rightleftharpoons [Ir^{III}]^+ + e^-$ — $23.06(E_{ox}([Ir^{II}]) = -59.3$ kcal mol^{-1} (Ref 29)
$[ColH]^+ \rightleftharpoons Col + H^+$ — $1.37(pK_a) = 20.6$ kcal mol^{-1} (Ref 22)
$\underline{H^+ + e^- \rightleftharpoons H^\bullet}$ — $\underline{C_G = 52.6 \text{ kcal mol}^{-1}}$
$HEH_2^* + [ColH]^+ \rightleftharpoons HEH_2^{\bullet+} + Col + H^\bullet$ — $BDFE_{eff}$ = **13.9 kcal mol^{-1}**

Estimation of $BDFE_{eff}$ for $[Ir^{III}(ppy)_3]^*$ as reductant and $[ColH]^+$ as acid:

$[Ir^{II}] \rightleftharpoons [Ir^{III}]^+ + e^-$ — $23.06(E_{ox}([Ir^{III*}]) = -48.7$ kcal mol^{-1} (Ref 29)
$[ColH]^+ \rightleftharpoons Col + H^+$ — $1.37(pK_a) = 20.6$ kcal mol^{-1} (Ref 22)
$\underline{H^+ + e^- \rightleftharpoons H^\bullet}$ — $\underline{C_G = 52.6 \text{ kcal mol}^{-1}}$
$HEH_2^* + [ColH]^+ \rightleftharpoons HEH_2^{\bullet+} + Col + H^\bullet$ — $BDFE_{eff}$ = **24.5 kcal mol^{-1}**

S8.3 Estimation of overpotential for hydrogenation of N_2 with HEH_2 to NH_3

We derive the BDFE of H_2 in MeCN at 298 K explicitly here for clarity, using recently updated thermochemical values (*36*):

$H_{2(g)} \rightleftharpoons 2\ H^+ + 2\ e^-$	$2\times23.06(E°(H^+/H_2)) = 2\times23.06(-0.028\ V) = -1.29\ kcal\ mol^{-1}$ (Ref 36)
$2\ H^+ + 2\ e^- \rightleftharpoons 2H^•$	$2(C_G) = 105.2\ kcal\ mol^{-1}$
$H_{2(g)} \rightleftharpoons 2H^•$	$BDFE(H_2) =$ **$103.9\ kcal\ mol^{-1}$**

S8.3.1 Determination of overpotential for dark reactions

The overpotential $\Delta\Delta G_f(NH_3)$ for a source of hydrogen atom equivalents with a given $BDFE_{eff}$ is described by **eqn S1**:

$$\Delta\Delta G_f(NH_3) = 3(BDFE(H_2)/2 - BDFE_{eff}) \quad \textbf{(eqn S1)}$$

For the dark reaction, $\frac{1}{2}\ N_2 + \frac{3}{2}\ HEH_2 \rightleftharpoons NH_3 + \frac{3}{2}\ HE$:

$$\Delta\Delta G_f(NH_3) = 3(103.9/2 - 51.4) = \mathbf{1.7\ kcal\ mol^{-1}}$$

For the dark reaction, $\frac{1}{2}\ N_2 + \frac{3}{2}\ acrH_2 \rightleftharpoons NH_3 + \frac{3}{2}\ acr$:

$$\Delta\Delta G_f(NH_3) = 3(103.9/2 - 59.9) = \mathbf{-23.9\ kcal\ mol^{-1}}$$

For the dark reaction, $\frac{1}{2}\ N_2 + \frac{3}{2}\ phenH_2 \rightleftharpoons NH_3 + \frac{3}{2}\ phen$:

$$\Delta\Delta G_f(NH_3) = 3(103.9/2 - 52.2) = \mathbf{-0.8\ kcal\ mol^{-1}}$$

For the dark reaction, $\frac{1}{2}\ N_2 + \frac{3}{2}\ (BNAH + ColH^+) \rightleftharpoons NH_3 + \frac{3}{2}\ (BNA^+ + Col)$:

$$\Delta\Delta G_f(NH_3) = 3(103.9/2 - 53.8) = \mathbf{-5.5\ kcal\ mol^{-1}}$$

With the dark reaction values value, we can also estimate the absolute driving force for hydrogenation of N_2 with $subH_2$.

$\Delta G_f(NH_3)$ in MeCN at 298 K:

$\frac{1}{2}\ N_2 + \frac{3}{2}\ H_{2(g)} \rightleftharpoons NH_3 \quad \Delta G_f(NH_3) = -3\times23.06(E°(N_{2(g)}/NH_3)) = -3\times23.06(0.063\ V\ vs.\ H_2)$ (Ref 36)
$= \mathbf{4.36\ kcal\ mol^{-1}}$

We then estimate the following:

$\frac{1}{2}\ N_2 + \frac{3}{2}\ HEH_2 \rightleftharpoons NH_3 + \frac{3}{2}\ HE \quad \Delta G_{rxn} = \Delta G_f(NH_3) - \Delta\Delta G_f(NH_3, HEH_2) = -4.4 - 1.7\ kcal\ mol^{-1}$
$= \mathbf{-6.1\ kcal\ mol^{-1}}$

$\frac{1}{2}\ N_2 + \frac{3}{2}\ phenH_2 \rightleftharpoons NH_3 + \frac{3}{2}\ phen \quad \Delta G_{rxn} = \Delta G_f(NH_3) - \Delta\Delta G_f(NH_3, phenH_2) = -4.4 + 0.8\ kcal\ mol^{-1}$
$= \mathbf{-3.6\ kcal\ mol^{-1}}$

$\frac{1}{2}\ N_2 + \frac{3}{2}\ acrH_2 \rightleftharpoons NH_3 + \frac{3}{2}\ acr\ \Delta G_{rxn} = \Delta G_f(NH_3) - \Delta\Delta G_f(NH_3, acrH_2) = -4.4 + 23.9\ kcal\ mol^{-1}$

$= \mathbf{19.5\ kcal\ mol^{-1}}$

$\frac{1}{2}\ N_2 + \frac{3}{2}\ (BNAH + ColH^+) \rightleftharpoons NH_3 + \frac{3}{2}\ (BNA^+ + Col)$

$\Delta G_{rxn} = \Delta G_f(NH_3) - \Delta\Delta G_f(NH_3, BNAH + ColH^+) = -4.4 + 5.5\ kcal\ mol^{-1}$

$= \mathbf{+1.1\ kcal\ mol^{-1}}$

**$subH_2$**	**$BDFE_{eff}$ (kcal mol^{-1})**	**$\Delta\Delta G_f(NH_3)$ (kcal mol^{-1})**	**ΔG_{rxn} (kcal mol^{-1})**
HEH_2	51.4	1.7	−6.1
$PhenH_2$	52.2	−0.8	−3.6
$AcrH_2$	59.9	−23.9	19.5
BNAH + [ColH]OTf [a]	53.8	−5.5	1.1

Table S10. Summary of driving forces for dark reaction using different $subH_2$ (assuming 2 H^+/ 2 e^- reaction). Values are calculated in MeCN at 25 °C. [a] Since BNAH is a H^- donor the stoichiometry requires addition iof H^+, assumed to be supplied from [ColH]OTf

S8.3.2 Determination of overpotential for light reactions

For the Ir-free reaction, in which $[HEH_2]^*$ is thought to be the strongest reductant accessed and $[ColH]^+$ serves as acid:

$$\Delta\Delta G_f(NH_3) = 3(103.9/2 - 10.9) = \mathbf{123.2\ kcal\ mol^{-1}}$$

For the Ir-photosensitized reaction with $[Ir(ppy)_2(dtbbpy)]BAr^F_4$, in which $[Ir^{II}]$ is thought to be the strongest reductant accessed and $[ColH]^+$ serves as acid:

$$\Delta\Delta G_f(NH_3) = 3(103.9/2 - 29.3) = \mathbf{68.0\ kcal\ mol^{-1}}$$

For the Ir-photosensitized reaction with $[Ir(dF(CF_3)ppy)_2(dtbbpy)]PF_6$, in which $[Ir^{II}]$ is thought to be the strongest reductant accessed and $[ColH]^+$ serves as acid:

$$\Delta\Delta G_f(NH_3) = 3(103.9/2 - 32.8) = \mathbf{58.3\ kcal\ mol^{-1}}$$

For the Ir-photosensitized reaction with $[Ir(p\text{-}F(Me)ppy)_2(dtbbpy)]PF_6$, in which $[Ir^{II}]$ is thought to be the strongest reductant accessed and $[ColH]^+$ serves as acid:

$$\Delta\Delta G_f(NH_3) = 3(103.9/2 - 29.8) = \mathbf{67.3\ kcal\ mol^{-1}}$$

For the Ir-photosensitized reaction with $Ir(ppy)_3$, in which $[Ir^{II}]$ is thought to be the strongest reductant accessed and $[ColH]^+$ serves as acid:

$$\Delta\Delta G_f(NH_3) = 3(103.9/2 - 13.9) = \mathbf{115.0\ kcal\ mol^{-1}}$$

For the Ir-photosensitized reaction with $Ir(ppy)_3$, in which $[Ir^{III}]^*$ is thought to be the strongest reductant accessed and $[ColH]^+$ serves as acid:

$$\Delta\Delta G_f(NH_3) = 3(103.9/2 - 24.5) = \mathbf{83.2\ kcal\ mol^{-1}}$$

Reductant	E_{ox} (V vs $Fc^{+/0}$)	$BDFE_{eff}$ (kcal mol^{-1})	$\Delta\Delta G_f(NH_3)$ (kcal mol^{-1})
HEH_2*	−2.70	10.9	123.2
$[Ir^{II}]$ ($Ir^{II}(ppy)_2$dtbbpy)	−1.90	29.3	68.0
$Ir^{II}(dF(CF_3)ppy)_2$(dtbbpy)	−1.75	32.8	58.3
Ir^{II}(*p*-F(Me)ppy)$_2$(dtbbpy)	−1.88	29.8	67.3
$Ir^{II}(ppy)_3^-$	−2.57	13.9	115.0
$Ir^{III}(ppy)_3$*	−2.11	24.5	83.2

Table S11: Summary of driving forces for different photosensitizers. All $BDFE_{eff}$ and $\Delta\Delta G_f(NH_3)$ measurements made pairing reductant with [ColH]OTf (pK_a 15) Values are calculated in MeCN at 25 °C.

S9 NMR spectra:

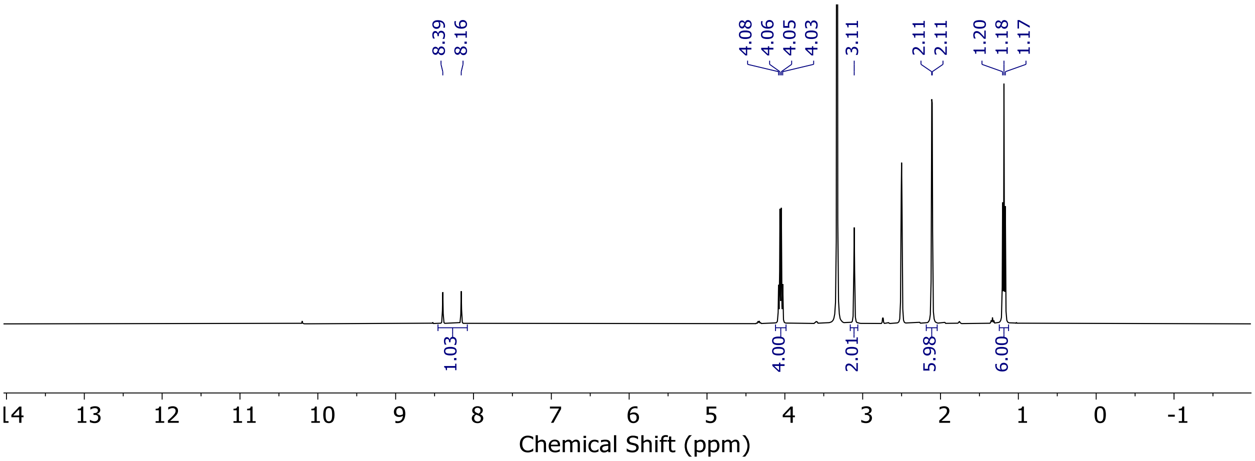

Figure S22. ^{1}H NMR (DMSO-d_6, 400 MHz) of ^{15}N-HEH$_2$.

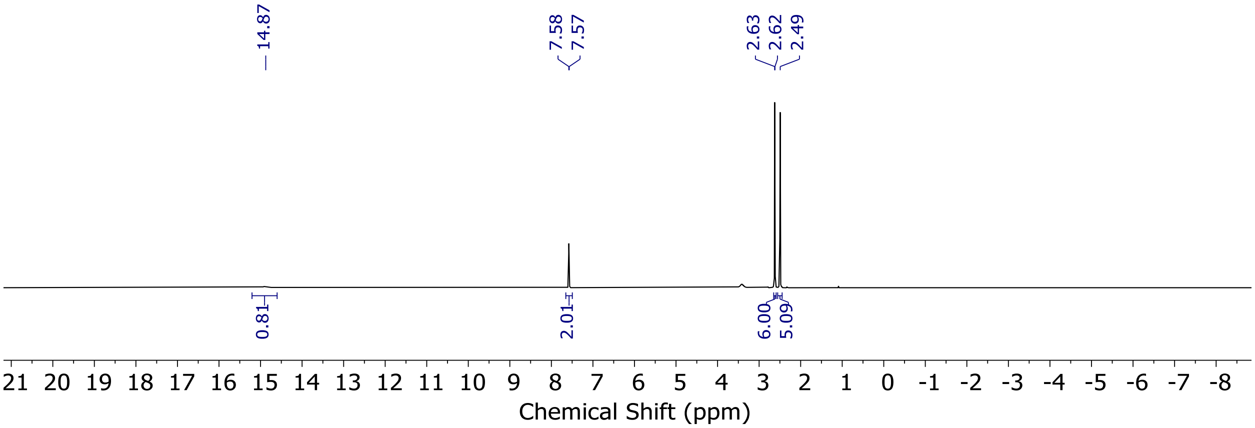

Figure S23. ^{1}H NMR (DMSO-d_6, 400 MHz) of ^{15}N-labelled [ColH]OTf.

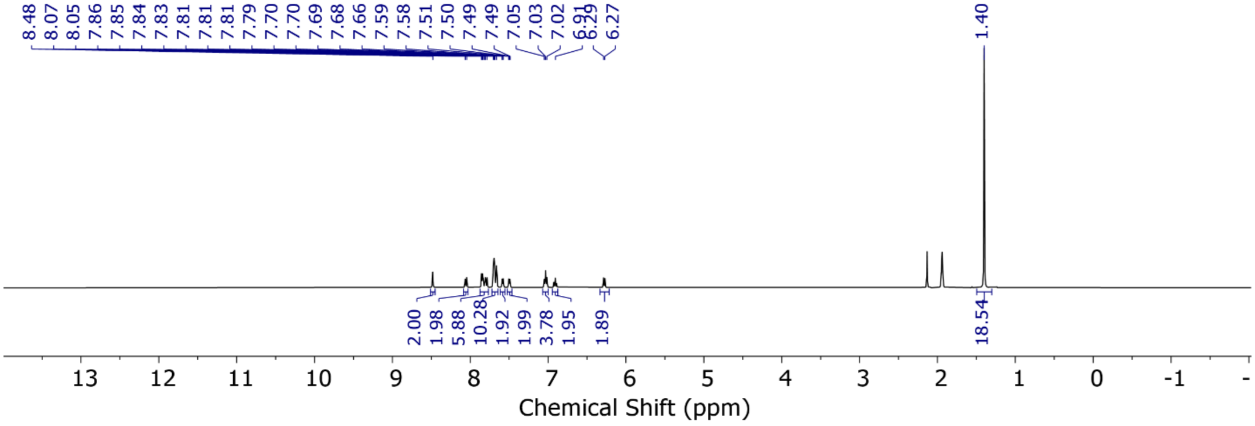

Figure S24. ^{1}H NMR (MeCN-d_3, 400 MHz) of [Ir]BAr$^{F}{}_4$.

REFERENCES AND NOTES

1. M. J. Chalkley, M. W. Drover, J. C. Peters, Catalytic N_2-to-NH_3 (or -N_2H_4) conversion by well-defined molecular coordination complexes. *Chem. Rev.* **120**, 5582–5636 (2020).

2. Y. Nishibayashi, Development of catalytic nitrogen fixation using transition metal–dinitrogen complexes under mild reaction conditions. *Dalton Trans.* **47**, 11290–11297 (2018).

3. R. R. Schrock, Catalytic reduction of dinitrogen to ammonia by molybdenum: Theory versus experiment. *Angew. Chem. Int. Ed.* **47**, 5512–5522 (2008).

4. L. C. Seefeldt, B. M. Hoffman, J. W. Peters, S. Raugei, D. N. Beratan, E. Antony, D. R. Dean, Energy transduction in nitrogenase. *Acc. Chem. Res.* **51**, 2179–2186 (2018).

5. V. Smil, *Enriching the Earth: Fritz Haber, Carl Bosch, and the Transformation of World Food Production* (MIT Press, 2000).

6. B. H. R. Suryanto, K. Matuszek, J. Choi, R. Y. Hodgetts, H.-L. Du, J. M. Bakker, C. S. M. Kang, P. V. Cherepanov, A. N. Simonov, D. R. MacFarlane, Nitrogen reduction to ammonia at high efficiency and rates based on a phosphonium proton shuttle. *Science* **372**, 1187 (2021), 1191.

7. Y. Yamazaki, H. Takeda, O. Ishitani, Photocatalytic reduction of CO_2 using metal complexes. *J. Photochem. Photobiol. C* **25**, 106–137 (2015).

8. N. Elgrishi, M. B. Chambers, X. Wang, M. Fontecave, Molecular polypyridine-based metal complexes as catalysts for the reduction of CO_2. *Chem. Soc. Rev.* **46**, 761–796 (2017).

9. P.-Z. Wang, J.-R. Chen, W.-J. Xiao, Hantzsch esters: An emerging versatile class of reagents in photoredox catalyzed organic synthesis. *Org. Biomol. Chem.* **17**, 6936–6951 (2019).

10. Q.-A. Chen, M.-W. Chen, C.-B. Yu, L. Shi, D.-S. Wang, Y. Yang, Y.-G. Zhou, Biomimetic asymmetric hydrogenation: In situ regenerable Hantzsch esters for asymmetric hydrogenation of benzoxazinones. *J. Am. Chem. Soc.* **133**, 16432–16435 (2011).

11. G.M. Abou-Elenein, N. A. Ismail, Z. F. Mohammed, H. M. Fahmy, Electroreduction of 2,6 dimethyl-3,4,5- trisubstituted pyridine derivatives in aqueous buffered media at carbon electrode. *Egypt. J. Pharmaceutical Sci.* **33**, 953–962 (1993).

12. B. M. Comer, P. Fuentes, C. O. Dimkpa, Y.-H. Liu, C. A. Fernandez, P. Arora, M. Realff, U. Singh, M. C. Hatzell, A. J. Medford, Prospects and challenges for solar fertilizers. *Joule* **3**, 1578–1605 (2019).

13. G. N. Schrauzer, T. D. Guth, Photolysis of water and photoreduction of nitrogen on titanium dioxide. *J. Am. Chem. Soc.* **99**, 7189–7193 (1977).

14. J. G. Edwards, J. A. Davies, D. L. Boucher, A. Mennad, An opinion on the heterogeneous photoreactions of N_2 with H_2O. *Angew. Chem. Int. Ed.* **31**, 480–482 (1992).

15. A. J. Medford, M. C. Hatzell, Photon-driven nitrogen fixation: Current progress, thermodynamic considerations, and future outlook. *ACS Catal.* **7**, 2624–2643 (2017).

16. K. A. Brown, D. F. Harris, M. B. Wilker, A. Rasmussen, N. Khadka, H. Hamby, S. Keable, G. Dukovic, J. W. Peters, L. C. Seefeldt, P. W. King, Light-driven dinitrogen reduction catalyzed by a CdS:nitrogenase MoFe protein biohybrid. *Science* **352**, 448–450 (2016).

17. K. A. Brown, J. Ruzicka, H. Kallas, B. Chica, D. W. Mulder, J. W. Peters, L. C. Seefeldt, G. Dukovic, P. W. King, Excitation-rate determines product stoichiometry in photochemical ammonia production by CdS quantum dot-nitrogenase MoFe protein complexes. *ACS Catal.* **10**, 11147–11152 (2020).

18. G.-B. Shen, Y.-H. Fu, X.-Q. Zhu, Thermodynamic network cards of Hantzsch ester, benzothiazoline, and dihydrophenanthridine releasing two hydrogen atoms or ions on 20 elementary steps. *J. Org. Chem.* **85**, 12535–12543 (2020).

19. J. Jung, J. Kim, G. Park, Y. You, E. J. Cho, Selective debromination and α-hydroxylation of α-bromo ketones using Hantzsch esters as photoreductants. *Adv. Synth. Catal.* **358**, 74–80 (2016)

20. D.-L. Zhu, Q. Wu, H.-Y. Li, H.-X. Li, J.-P. Lang, Hantzsch ester as a visible-light photoredox catalyst for transition-metal-free coupling of arylhalides and arylsulfinates. *Chem. Eur. J.* **26**, 3484–3488 (2020)

21. K. Arashiba, A. Eizawa, H. Tanaka, K. Nakajima, K. Yoshizawa, Y. Nishibayashi, Catalytic nitrogen fixation via direct cleavage of nitrogen–nitrogen triple bond of molecular dinitrogen under ambient reaction conditions. *Bull. Chem. Soc. Jpn.* **90**, 1111–1118 (2017).

22. S. Tshepelevitsh, A. Kütt, M. Lõkov, I. Kaljurand, J. Saame, A. Heering, P. G. Plieger, R. Vianello, I. Leito, On the basicity of organic bases in different media. *Eur. J. Org. Chem.* **2019**, 6735–6748 (2019).

23. N. G. Connelly, W. E. Geiger, Chemical redox agents for organometallic chemistry. *Chem. Rev.* **96**, 877–910 (1996).

24. K. Kano, T. Matsuo, Photoinduced one-electron reduction of 1-benzyl-3-carbamoylpyridinium chloride and 3,5-bis(ethoxycarbonyl)-2,6-dimethylpyridine. *Bull. Chem. Soc. Jpn.* **49**, 3269–3273 (1976).

25. T. Munisamy, R. R. Schrock, An electrochemical investigation of intermediates and processes involved in the catalytic reduction of dinitrogen by [HIPTN$_3$N]Mo (HIPTN$_3$N = (3,5-(2,4,6-i-Pr$_3$C$_6$H$_2$)$_2$C$_6$H$_3$NCH$_2$CH$_2$)$_3$N). *Dalton Trans.* **41**, 130–137 (2012).

26. H. Tanaka, K. Arashiba, S. Kuriyama, A. Sasada, K. Nakajima, K. Yoshizawa, Y. Nishibayashi, Unique behaviour of dinitrogen-bridged dimolybdenum complexes bearing pincer ligand towards catalytic formation of ammonia. *Nat. Commun.* **5**, 3737–3787 (2014).

27. D. Wang, F. Loose, P. J. Chirik, R. R. Knowles, N–H bond formation in a manganese(V) nitride yields ammonia by light-driven proton-coupled electron transfer. *J. Am. Chem. Soc.* **141**, 4795–4799 (2019).

28. J. D. Slinker, A. A. Gorodetsky, M. S. Lowry, J. Wang, S. Parker, R. Rohl, S. Bernhard, G. G. Malliaras, Efficient yellow electroluminescence from a single layer of a cyclometalated iridium complex. *J. Am. Chem. Soc.* **126**, 2763–2767 (2004).

29. T. Koike, M. Akita, Visible-light radical reaction designed by Ru- and Ir-based photoredox catalysis. *Inorg. Chem. Front.* **1**, 562–576 (2014).

30. A. G. Capacci, J. T. Malinowski, N. J. McAlpine, J. Kuhne, D. W. C. MacMillan, Direct, enantioselective α-alkylation of aldehydes using simple olefins. *Nat. Chem.* **9**, 1073–1077 (2017).

31. H. Hirakawa, M. Hashimoto, Y. Shiraishi, T. Hirai, Selective nitrate-to-ammonia transformation on surface defects of titanium dioxide photocatalysts. *ACS Catal.* **7**, 3713–3720 (2017).

32. A. H. Boonstra, C. A. H. A. Mutsaers, Photohydrogenation of ethyne and ethene on the surface of titanium dioxide. *J. Phys. Chem.* **79**, 2025–2027 (1975).

33. Y. Zhang, Y. Tanabe, S. Kuriyama, Y. Nishibayashi, Photoredox- and nickel-catalyzed hydroalkylation of alkynes with 4-alkyl-1,4-dihydropyridines: ligand-controlled regioselectivity. *Chem. Eur. J.* **28**, e202200727 (2022).

34. F. G. Bordwell, J. P. Cheng, J. A. Harrelson, Homolytic bond dissociation energies in solution from equilibrium acidity and electrochemical data. *J. Am. Chem. Soc.* **110**, 1229–1231 (1988).

35. M. Tilset, V. D. Parker, Solution homolytic bond dissociation energies of organotransition-metal hydrides. *J. Am. Chem. Soc.* **111**, 6711–6717 (1989).

36. R. G. Agarwal, S. C. Coste, B. D. Groff, A. M. Heuer, H. Noh, G. A. Parada, C. F. Wise, E. M. Nichols, J. J. Warren, J. M. Mayer, Free energies of proton-coupled electron transfer reagents and their applications. *Chem. Rev.* **122**, 1–49 (2022).

37. M. J. Chalkley, J. C. Peters, Relating N–H bond strengths to the overpotential for catalytic nitrogen fixation. *Eur. J. Inorg. Chem.* **2020**, 1353–1357 (2020).

38. B. E. Norcross, G. Clement, M. Weinstein, The Hantzsch pyridine synthesis: A factorial design experiment for the introductory organic laboratory. *J. Chem. Educ.* **46**, 694 (1969).

39. J. S. Anderson, M. E. Moret, J. C. Peters, Conversion of Fe–NH_2 to Fe–N_2 with release of NH_3. *J. Am. Chem. Soc.* **135**, 534–537 (2013).

40. T. J. Del Castillo, N. B. Thompson, J. C. Peters, A synthetic single-site Fe nitrogenase: High turnover, Freeze-Quench 57Fe Mössbauer data, and a hydride resting state. *J. Am. Chem. Soc.* **138**, 5341–5350 (2016).

41. N. S. Golubev, S. N. Smirnov, P. Schah-Mohammedi, I. G. Shenderovich, G. S. Denisov, V. A. Gindin, H. H. Limbach, Study of acid-base interaction by means of low-temperature NMR spectra. Structure of salicylic acid complexes. *Russ. J. Gen. Chem.* **67**, 1082–1087 (1997).

42. L. Matesic, J. M. Locke, K. L. Vine, M. Ranson, J. B. Bremner, D. Skropeta, Synthesis and anti-leukaemic activity of pyrrolo[3,2,1-hi]indole-1,2-diones, pyrrolo[3,2,1-ij]quinoline-1,2-diones and other polycyclic isatin derivatives. *Tetrahedron* **68**, 6810–6819 (2012).

43. R. Brišar, F. Unglaube, D. Hollmann, H. Jiao, E. Mejía, Aerobic oxidative homo- and cross-coupling of amines catalyzed by phenazine radical cations. *J. Org. Chem.* **83**, 13481–13490 (2018).

44. L. T. Elrod, E. Kim, Lewis acid assisted nitrate reduction with biomimetic molybdenum oxotransferase complex. *Inorg. Chem.* **57**, 2594–2602 (2018).

45. G. R. Fulmer, A. J. M. Miller, N. H. Sherden, H. E. Gottlieb, A. Nudelman, B. M. Stoltz, J. E. Bercaw, K. I. Goldberg, NMR chemical shifts of trace impurities: Common laboratory solvents, organics, and gases in deuterated solvents relevant to the organometallic chemist. *Organometallics* **29**, 2176–2179 (2010).

46. M. W. Weatherburn, Phenol-hypochlorite reaction for determination of ammonia. *Anal. Chem.* **39**, 971–974 (1967).

47. G. W. Watt, J. D. Chrisp, Spectrophotometric method for determination of hydrazine. *Anal. Chem.* **24**, 2006–2008 (1952).

48. F. Gibanel, M. C. López, F. M. Royo, J. Santafé, J. S. Urieta, Solubility of nonpolar gases in tetrahydrofuran at 0 to 30°C and 101.33 kPa partial pressure of gas. *J. Solution Chem.* **22**, 211–217 (1993).

49. S. Xu, D. C. Ashley, H.-Y. Kwon, G. R. Ware, C.-H. Chen, Y. Losovyj, X. Gao, E. Jakubikova, J. M. Smith, A flexible, redox-active macrocycle enables the electrocatalytic reduction of nitrate to ammonia by a cobalt complex. *Chem. Sci.* **9**, 4950–4958 (2018).

50. B. M. Lindley, A. M. Appel, K. Krogh-Jespersen, J. M. Mayer, A. J. M. Miller, Evaluating the thermodynamics of electrocatalytic N_2 reduction in acetonitrile. *ACS Energy Lett.* **1**, 698–704 (2016).

51. X.-Q. Zhu, H.-R. Li, Q. Li, T. Ai, J.-Y. Lu, Y. Yang, J.-P. Cheng, Determination of the C4−H bond dissociation energies of NADH models and their radical cations in acetonitrile. *Chem. Eur. J.* **9**, 871–880 (2003).

52. E. S. Wiedner, M. B. Chambers, C. L. Pitman, R. M. Bullock, A. J. M. Miller, A. M. Appel, Thermodynamic hydricity of transition metal hydrides. *Chem. Rev.* **116**, 8655–8692 (2016).

53. S. Ilic, U. Pandey Kadel, Y. Basdogan, J. A. Keith, K. D. Glusac, Thermodynamic hydricities of biomimetic organic hydride donors. *J. Am. Chem. Soc.* **140**, 4569–4579 (2018).
